# Estimation of relative biological effectiveness of ^225^Ac compared to ^177^Lu during [^225^Ac]Ac-PSMA and [^177^Lu]Lu-PSMA radiopharmaceutical therapy using TOPAS/TOPAS-nBio/MEDRAS

**DOI:** 10.1186/s40658-023-00567-2

**Published:** 2023-09-11

**Authors:** Mikhail Rumiantcev, Wei Bo Li, Simon Lindner, Grigory Liubchenko, Sandra Resch, Peter Bartenstein, Sibylle I. Ziegler, Guido Böning, Astrid Delker

**Affiliations:** 1 Department of Nuclear Medicine, LMU University Hospital, LMU Munich, Munich, Germany; 2 Federal Office for Radiation Protection, Medical and Occupational Radiation Protection, Oberschleißheim, Germany

**Keywords:** mCRPC, Radiopharmaceutical therapy, [^225^Ac]Ac-PSMA, [^177^Lu]Lu-PSMA, RBE

## Abstract

**Aim:**

Over recent years, [^225^Ac]Ac-PSMA and [^177^Lu]Lu-PSMA radiopharmaceutical therapy have evolved as a promising treatment option for advanced prostate cancer. Especially for alpha particle emitter treatments, there is still a need for improving dosimetry, which requires accurate values of relative biological effectiveness (RBE). To achieve that, consideration of DNA damages in the cell nucleus and knowledge of the energy deposition in the location of the DNA at the nanometer scale are required. Monte Carlo particle track structure simulations provide access to interactions at this level. The aim of this study was to estimate the RBE of ^225^Ac compared to ^177^Lu. The initial damage distribution after radionuclide decay and the residual damage after DNA repair were considered.

**Methods:**

This study employed the TOol for PArtcile Simulation (TOPAS) based on the Geant4 simulation toolkit. Simulation of the nuclear DNA and damage scoring were performed using the TOPAS-nBio extension of TOPAS. DNA repair was modeled utilizing the Python-based program MEDRAS (Mechanistic DNA Repair and Survival). Five different cell geometries of equal volume and two radionuclide internalization assumptions as well as two cell arrangement scenarios were investigated. The radionuclide activity (number of source points) was adopted based on SPECT images of patients undergoing the above-mentioned therapies.

**Results:**

Based on the simulated dose–effect curves, the RBE of ^225^Ac compared to ^177^Lu was determined in a wide range of absorbed doses to the nucleus. In the case of spherical geometry, 3D cell arrangement and full radionuclide internalization, the RBE based on the initial damage had a constant value of approximately 2.14. Accounting for damage repair resulted in RBE values ranging between 9.38 and 1.46 for ^225^Ac absorbed doses to the nucleus between 0 and 50 Gy, respectively.

**Conclusion:**

In this work, the consideration of DNA repair of the damage from [^225^Ac]Ac-PSMA and [^177^Lu]Lu-PSMA revealed a dose dependency of the RBE. Hence, this work suggested that DNA repair is an important aspect to understand response to different radiation qualities.

**Supplementary Information:**

The online version contains supplementary material available at 10.1186/s40658-023-00567-2.

## Introduction

Prostate cancer is one of the main causes of cancer-related mortality among males [1]. The treatment of patients with metastatic castration-resistant prostate cancer (mCRPC) is based on targeting the prostate-specific membrane antigen (PSMA), which is a transmembrane glycoprotein that is overexpressed in malignant prostate cancer cells [2]. Being labeled with radionuclides that are confined in chelators, PSMA ligands selectively bind to PSMA and are then internalized by endocytosis into the tumor cells [3]. The particles emitted by the radionuclides may deposit their energy locally and eventually mediate cell death. The radiobiological effect of the treatment is caused by the induction of DNA damages in the cell nucleus. [^177^Lu]Lu-PSMA therapy of patients with mCRPC represents a well-established therapy option under usage of $\beta^{ - }$-emitting ^177^Lu [4]. However, there is a fraction of patients at an advanced stage of mCRPC who become resistant to [^177^Lu]Lu-PSMA therapy [5]. In this case, [^225^Ac]Ac-PSMA therapy, which utilizes α-emitting ^225^Ac, comes into use. Alpha particles represent a radiation quality with a high linear energy transfer (LET),which leads to a higher value of the relative biological effectiveness (RBE) compared to electrons and which is beneficial for cell killing. According to Sgouros et al. [6], when performing dosimetry for deterministics effects as it is the case for radionuclide therapy, the absorbed dose to organ or lesion should be weighted by an appropriate value of RBE. For alpha particles emitted from ^225^Ac, an RBE value of 5 is used for an estimate of the absorbed dose in clinical practice [5]. It was recommended by Sgouros et al. [6] based on a report of a meeting conducted by the United States Department of Energy in 1996 [7]. The variations in therapy response with regard to estimated doses may suggest an adaption of this RBE value. As stated in the work of Sgouros et al. [6], an RBE value of between 3 and 5 was recommended for α-particle emitters for cell killing based on experimental data; the proposed value of 5 was intended to project the possible deterministic biological effects associated with an estimated absorbed dose. Furthermore, Feinendegen et al. [7] reported that an RBE value of 5 could be considered as an initial value based on limited data and might need to be changed with increasing number of clinical trials. The currently utilized RBE value of 5 does not account for the individual characteristic alpha particle energy of different radionuclides. Kratochwil et al. [5] hypothesized that an RBE value of 5 might be overcautious regarding bone marrow toxicity, whereas it might underestimate salivary gland toxicity. Hobbs et al. [8] have shown that in the scope of alpha-emitter therapy, RBE is dose-dependent and thus the specification of RBE requires a specification of the absorbed dose. In concordance, we assume that tissue dosimetry for [^225^Ac]Ac-PSMA therapy would greatly benefit from an improvement of underlying RBE values.

Thus, the aim of this study was to investigate the RBE of ^225^Ac compared to ^177^Lu during [^225^Ac]Ac-PSMA and [^177^Lu]Lu-PSMA therapy by means of event-by-event Monte Carlo particle track structure simulations that provide access to the energy deposition in the location of DNA at the nanometer scale. The RBE of ^225^Ac could then be used to determine the RBE-weighted doses according to the MIRD pamphlet no. 22 [6] or to estimate the activity of ^225^Ac required to achieve the same biological effect as in the case of [^177^Lu]Lu-PSMA therapy from the absorbed dose in the case of [^177^Lu]Lu-PSMA therapy.

As mentioned by Li et al. [9] in a review on micro- and nanodosimetry for internal emitters, there are several well-developed Monte Carlo programs which are mostly used in the community of track structure calculations and nanodosimetry, e.g., PARTRAC (Friedland et al. [10]), Geant4-DNA (Incerti et al. [11]), PENELOPE (Salvat [12]), NASIC (Li et al. [13]). We used the TOol for PArtcile Simulation (TOPAS, version 3.7.p1) [14, 15] based on the Geant4 simulation toolkit (version geant4-10–06-patch-03) [16–18]. In brief, TOPAS wraps and extends Geant4 C++ classes providing a ready-to-use simulation platform. Being layered on top of Geant4, which has been extensively validated in different applications including medical physics [19–26], TOPAS is a well-validated tool. To simulate the interactions of electrons and alpha particles with the nuclear DNA, the TOPAS-nBio extension to TOPAS (version TOPAS-nBio-1.0) [27] was employed, which incorporates the processes of the Geant4-DNA extension [28–31] to the general purpose Monte Carlo toolkit Geant4. Geant4-DNA has been designed for modeling of biological damage induced by ionizing radiation at the DNA scale. TOPAS-nBio has been carefully validated and evaluated in radiobiological studies simulating DNA damages and water radiolysis for gamma, proton and alpha particle irradiations [32–42].

## Methods

In 79.3% of events, ^177^Lu decays by $\beta^{ - }$-decay to the ground state of ^177^Hf with a maximum beta energy $E_{{\beta^{ - } , \max }}$ of 498 keV. In 11.58% of beta transitions ($E_{{\beta^{ - } , \max }} = 177\;{\text{keV}}$), ^177^Lu disintegrates to an excited state of ^177^Hf that lies 321 keV above the ground state. During the remaining 9.1% of disintegrations ($E_{{\beta^{ - } , \max }} = 385 \;{\text{keV}}$), ^177^Lu decays to an excited state of ^177^Hf with an energy of 113 keV. The excited states are mainly depopulated by gamma transition. The energy of an excited nucleus can be also transferred to an orbital electron (internal conversion), leaving a vacancy which is filled by an electron from a higher energy level, resulting in emission of characteristic X-rays or Auger electrons. Therefore, the electron spectrum of ^177^Lu also contains internal conversion electrons and Auger electrons. ^177^Lu has a half-life of 6.67 d [43].

The decay scheme of ^225^Ac is more complex. In brief, ^225^Ac decays to stable ^209^Bi ($T_{1_{/_{2}}} = 2.01 \times 10^{19} y$). This transition includes five $\alpha$-decays ($E_{\alpha }$ of 5.8 MeV, 6.3 MeV, 7.1 MeV, 5.9 MeV and 8.4 MeV) and three $\beta^{ - }$-decays ($E_{{\beta^{ - } , \max }}$ of 1.4 MeV, 2.0 MeV, 0.6 MeV). The half-life of ^225^Ac is 9.92 d [44].

The radioactive decay of ^177^Lu and ^225^Ac, the event-by-event transport of electrons and alpha particles in the cells and the surrounding medium (water) as well as water radiolysis, diffusion of radiolytic products, interactions of chemical species and induction of DNA damage were simulated using TOPAS/TOPAS-nBio. The subsequent DNA repair of the induced DNA damage was modeled utilizing the Python-based program MEDRAS (Mechanistic DNA Repair and Survival). The details providing insight into the utilized physics and chemistry modules, nucleus model, DNA damage scoring and DNA repair mechanism are described in the Additional file 1.

### Simulation setup

#### Simulation geometry

**Fig. 1:**
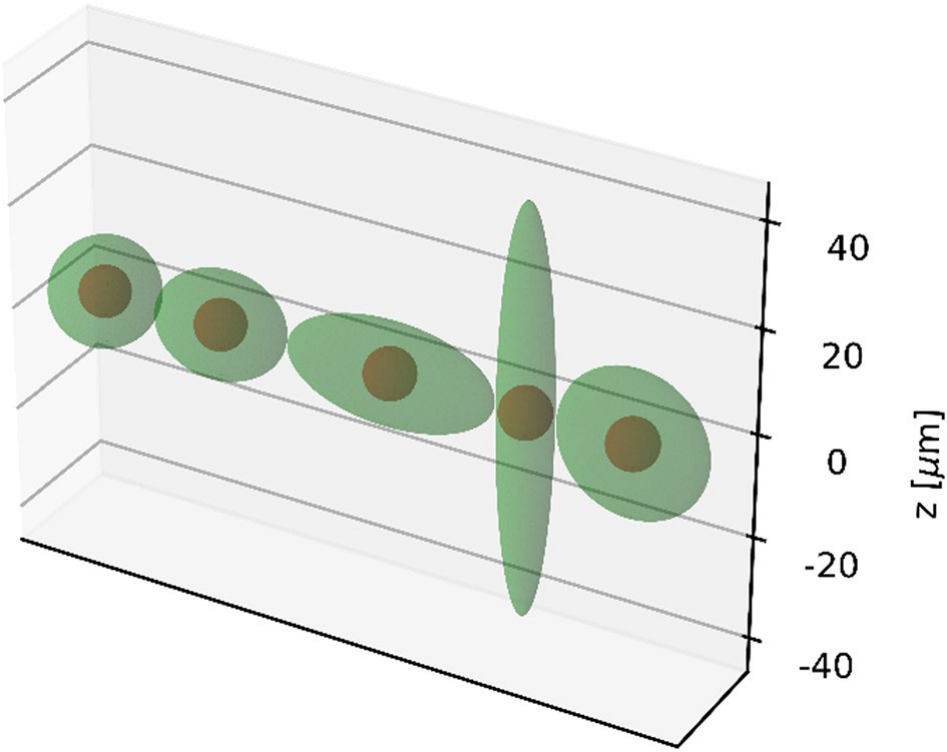
Cell geometries used in this work. See Table 1 for details

**Fig. 2:**
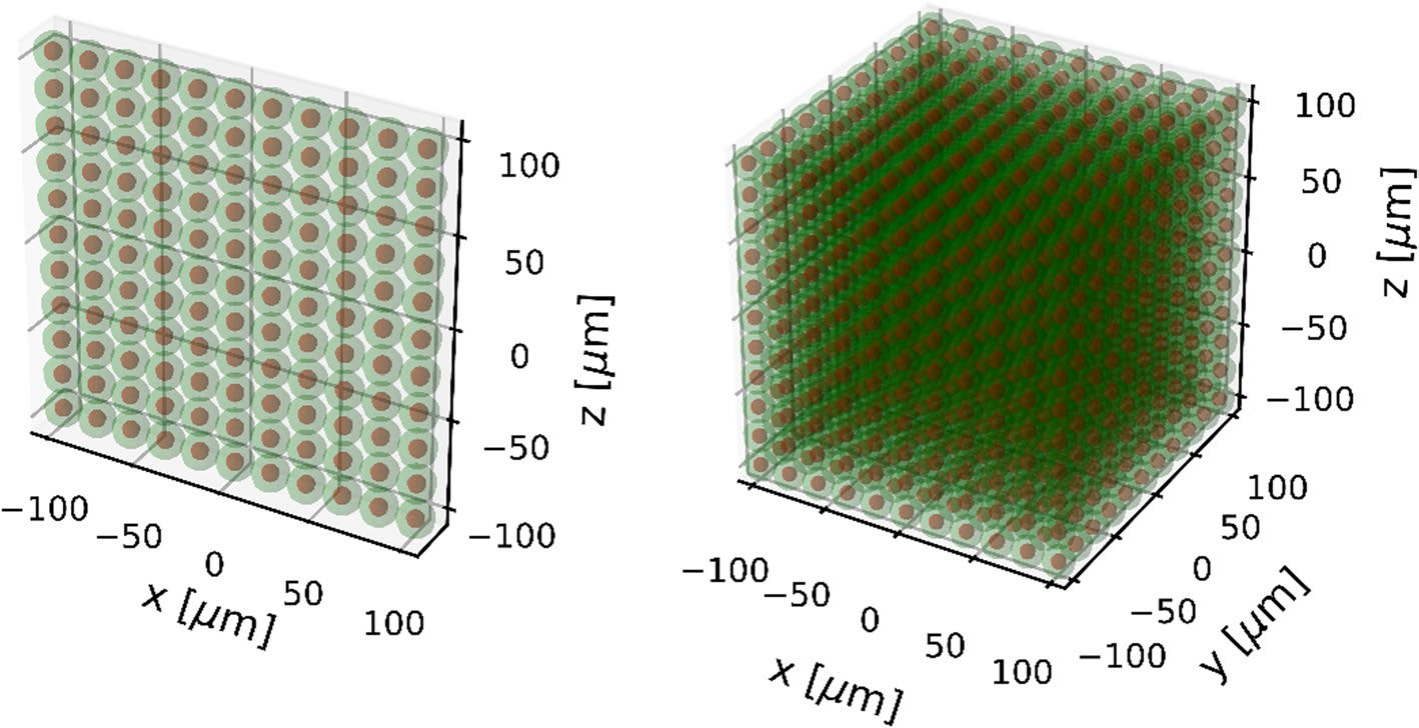
Cell arrangement scenarios. Example for ^225^Ac and cell geometry 1

Cells were assumed to be ellipsoids consisting of a membrane with a thickness of 10 nm, a cytoplasm and a spherical nucleus with a radius of 4.65 µm. The cell material was set to liquid water. Five different cell geometries described in Table 1 of equal volume of approx. 4189 µm^3^ were used. The cell geometries are shown in Fig. 1. Two cell arrangement scenarios were applied: two-dimensional and three-dimensional. The 3D arrangement was used to simulate the interactions of particles within a geometry that is closer to what is expected in a real therapy situation. The 2D arrangement was implemented to simulate the irradiation of cells as it might be the case in cell experiments. Such an experimental setup might be cell cultures that are grown to investigate different biological endpoints (DSBs, chromosomal aberrations, cell survival) caused by irradiation. The total number of cells to be simulated was chosen based on the range of electrons in the case of ^177^Lu irradiation and the range of alpha particles in the case of ^225^Ac irradiation. According to [45], the CSDA (continuous-slowing-down approximation) range of electrons with an energy of 0.5 MeV in liquid water is approx. 1.766 mm and the CSDA range of alpha particles with $E_{\alpha } = 8.5 \,{\text{MeV}}$ in liquid water is about 86.53 µm. As reported in [46], in the case of ^177^Lu, it is sufficient to take the CSDA range at the average electron energy (280 µm) instead of the CSDA range at the maximum electron energy, since the cross absorbed dose (i.e., the absorbed dose delivered by surrounding cells to a target cell) decays exponentially with the cell distance and the contribution of cells outside the CSDA range corresponding to the average electron energy would be negligible. In order to reduce the computational cost associated with the large number of cells to be simulated in the case of 3D cell arrangement for ^177^Lu, the half CSDA range at the average electron energy (140 µm) was used. The surrounding cells were placed in space around the central cell forming a rectangular cuboid. The number of cells along the x, y and z axes was calculated using the following formula:

1 $$n_{i} = 2 \times \biggl \lceil 0.5 \times \frac{{{\text{particle}}\;{\text{ range}} + R_{{{\text{nucleus}}}} }}{{{\text{HL}}_{i} }} - 1 \biggr \rceil + 1$$

where $i$ denotes x, y or z, ${\text{HL}}_{i}$ is the half-length of the ellipsoid along the corresponding axis and the square brackets indicate the ceiling function. The number of simulated cells for each geometry can be found in Table 2. Figure 2 illustrates the described cell arrangement scenarios.

**Table 1:** Half-length (HL) of the principal axes of ellispoidal cells used in this work

Cell geometry	HLX, µm	HLY, µm	HLZ, µm
1	10	10	10
2	12.5	8	10
3	20	5	10
4	5	5	40
5	14.142	5	14.142

**Table 2:** Number of cells simulated in this work

Cell geometry	Number of cells			
	^177^Lu		^225^Ac	
	2D	3D	2D	3D
1	29 × 29 = 841	15 × 15 × 15 = 3375	11 × 11 = 121	11 × 11 × 11 = 1331
2	23 × 29 = 667	13 × 15 × 19 = 3705	9 × 11 = 99	9 × 11 × 13 = 1287
3	15 × 29 = 435	9 × 15 × 29 = 3915	5 × 11 = 55	5 × 11 × 19 = 1045
4	57 × 9 = 513	29 × 5 × 29 = 4205	19 × 3 = 57	19 × 3 × 19 = 1083
5	21 × 21 = 44	11 × 11 × 29 = 3509	7 × 7 = 49	7 × 7 × 19 = 931

#### Source points distribution

The number of source points was adopted on the basis of SPECT images of patients undergoing [^177^Lu]Lu-PSMA-I&T or [^177^Lu]Lu-PSMA-617 therapy. The earliest time point (24 h) of SPECT imaging after therapy was used to determine the activity concentrations in lesions and no excretion of activity from tumor cells was considered. A total of 33 lesions treated with [^177^Lu]Lu-PSMA-I&T and 13 lesions treated with [^177^Lu]Lu-PSMA-617 with activity concentrations and volumes obtained from segmented SPECT images was used. These data originate from the work by Resch et al. [47]. For each lesion, the activity concentration at 24 h post-treatment per lesion was normalized to the corresponding injected activity and converted to the number of source points per cell $N_{{\text{cell, norm}}}$ using the relation $A = \frac{\ln 2}{T_{1_{/_{2}}}}  \times N$ between activity $A$ and number of radioactive particles $N$:

2 $$\begin{aligned} N_{{\rm{cell, norm}}}  [{{\text{per GBq}} }]  & = {\frac{T_{1/2}} {\ln 2}} \times {A_{{\rm{cell, norm}}}  [{Bq\,  {\text{per GBq}} }] } \\ & = {\frac{T_{1/2}}{\ln 2}} \times {1000} \times {{\text{norm. activity conc.}}} \\ &\quad \left[ {{\text{kBq mL}}^{ - 1} {\text{per GBq}}} \right] \times {{V_{{{\rm{cell}}}}}  [{{\text{mL}}} }] \end{aligned}$$

**Fig. 3:**
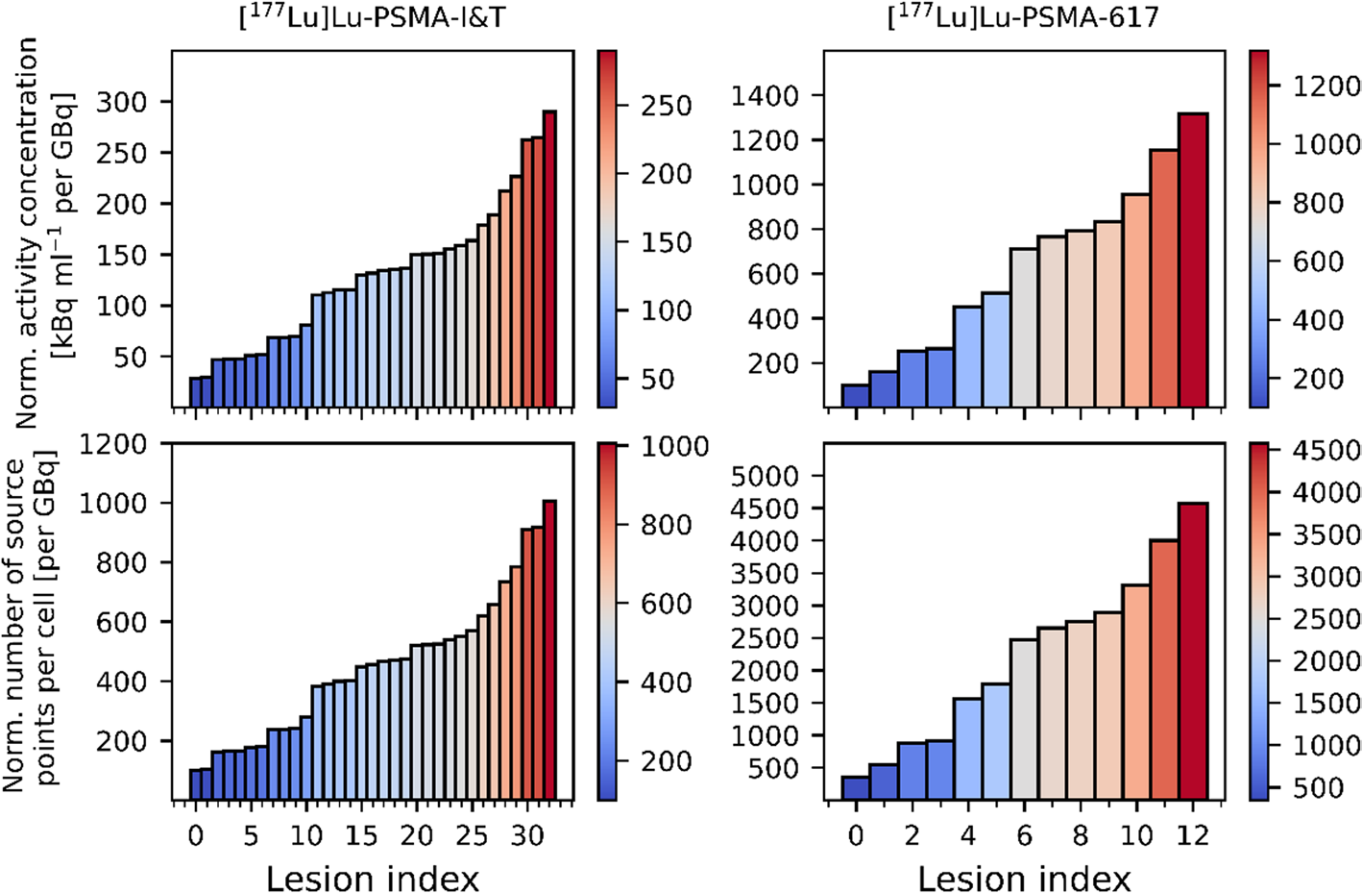
Normalized activity concentration per lesion and normalized number of source points per cell for ^177^Lu

In Eq. 2, $T_{1_{/_{2}}}$ denotes the physical half-life. Radionuclide release from the cells (excretion) is not accounted for. In Fig. 3, the normalized activity concentration per lesion and the normalized number of source points per cell for ^177^Lu are shown. The corresponding quantities for ^225^Ac were calculated by scaling the normalized activity concentration in lesion and the normalized number of source points per cell for ^177^Lu by a factor of

$$\frac{{8 {\text{ MBq}} \times 9.92 \text{ d}}}{{7400 {\text{ MBq}} \times 6.647 \text{ d} }} \approx 619.8$$

**Fig. 4:**
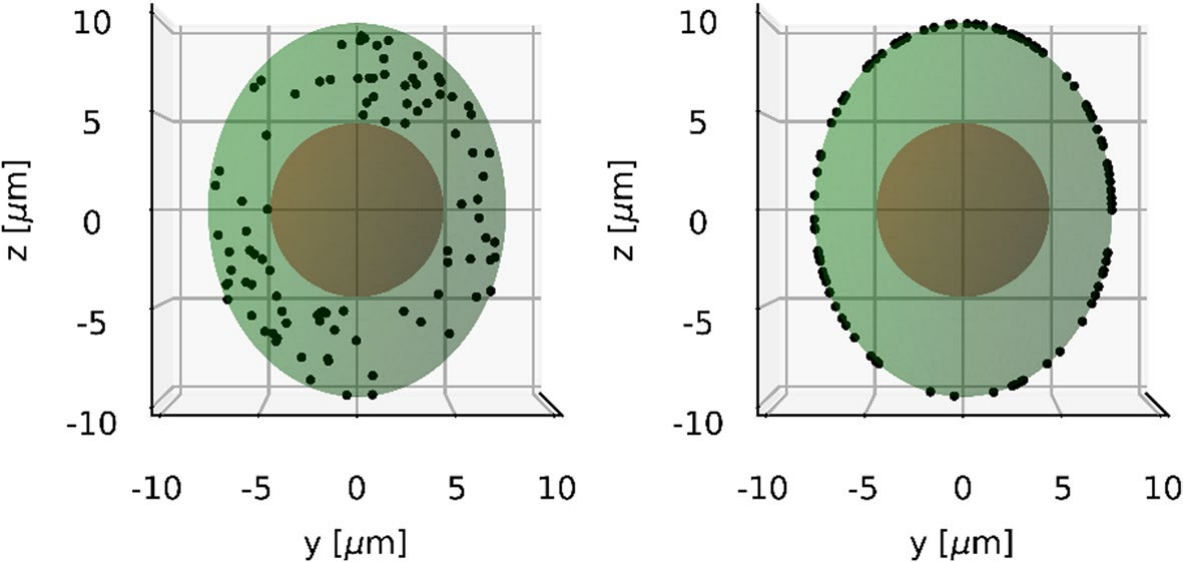
Internalization scenarios. An example for cell geometry 2

This is due to the fact that the ^177^Lu and ^225^Ac activity typically used in routine clinical practice is 7400 MBq (200 mCi) and 8 MBq (approx. 0.2162 mCi), respectively [48]. Based on the data from Fig. 3, the number of ^177^Lu radionuclides distributed in each cell was varied between 100, 300, 500, 700, 900, 1000, 2000, 3000, 4000 and 5000 to account for the calculated normalized numbers of source points in case of [^177^Lu]Lu-PSMA-I&T or [^177^Lu]Lu-PSMA-617. The corresponding numbers of ^225^Ac sources per cell were calculated by dividing the above-mentioned numbers by 619.8 and set to 1, 2, 3, 4, 5, 6, 7, 8, 9 and 10. For each cell geometry, the radionuclide sources were distributed either on the cell membrane (membrane-bound) or in the cytoplasm (fully internalized), which is depicted in Fig. 4.

### Statistics

For both ^177^Lu and ^225^Ac, each cell geometry, each cell arrangement and internalization scenario and each number of source points per cell, the simulation was run 10 times to achieve 10 independent runs with different seeds. Considering two different radionuclides, five different cell geometries, two different cell arrangement scenarios, two different internalization scenarios and ten different numbers of source points per cell, a total of 400 different simulation setups was investigated. 400 different simulations setups with ten histories per simulation setup resulted in a total of 4000 simulations that were run. For both ^177^Lu and ^225^Ac, for each cell geometry, each cell arrangement and internalization scenario, the simulated dose–effect curve $N_{{{\text{DSB}}}} = N_{{{\text{DSB}}}} \left( D \right)$ consists of 100 data points (ten different numbers of source points per cell with ten histories each). For each TOPAS simulation, the DNA repair simulation was performed 10 times to better describe the stochastic rejoining of free DNA ends.

### Relative biological effectiveness

In the following, the definition of the relative biological effectiveness (RBE) is addressed. RBE is used to compare the biological effect of two types of radiation. This term originates from [49]. According to [6], RBE is defined as the ratio of the absorbed dose of a reference radiation type required for a given biological effect and the absorbed dose of the radiation type under investigation for the same biological effect under identical experimental conditions.

3 $${\text{RBE}} = \left. {\frac{{D_{{{\text{ref}}}} }}{{D_{{{\text{investigated}}}} }}} \right|_{{{\text{isoeffect}}}}$$

**Fig. 5:**
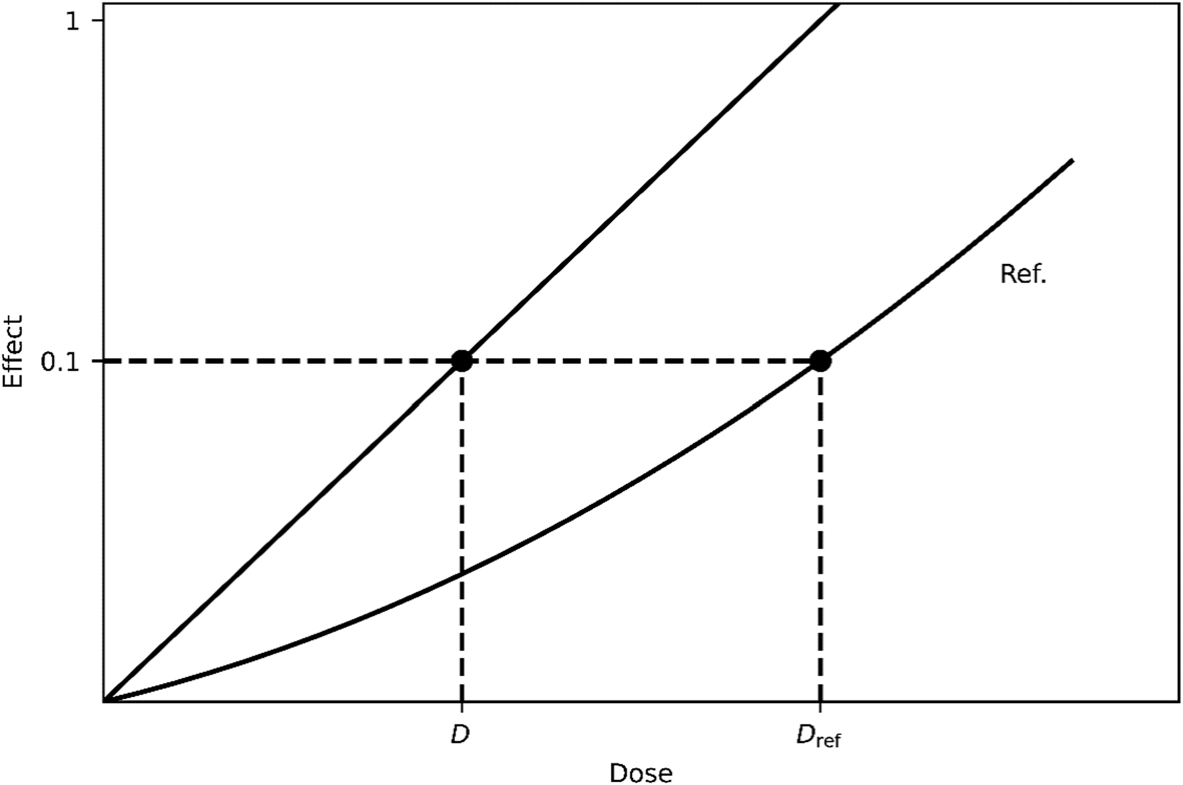
Determination of RBE according to the definition

RBE can be determined from cell survival curves or dose–effect curves. Figure 5 illustrates the determination of RBE according to the definition. In this work, the number of DSBs is considered as a biological effect of radiation and ^177^Lu serves as a reference radiation type.

For each simulation, the number of DSBs $N_{{{\text{DSB}}}}$ before and after DNA repair was plotted as a function of the absorbed dose to the nucleus $D$. The number of DSBs after DNA repair was calculated as the sum of residual and misrepaired DSBs. To determine the RBE from these data, the functions $N_{{{\text{DSB}}}} = N_{{{\text{DSB}}}} \left( D \right)$ were fitted. For ^177^Lu, a linear-quadratic relationship was used for fitting and for ^225^Ac, $N_{{{\text{DSB}}}} \left( D \right)$ was linearly fitted. The motivation to assume these relationships is addressed in Results and Discussion. Because there are no DSBs in the nucleus at $D = 0 \text{ Gy}$, $N_{{{\text{DSB}}}}$ for ^177^Lu and ^225^Ac can be written as follows:

4 $$N_{{{\text{DSB}}, {}_{{}}^{177} {\text{Lu}}}} \left( {D_{{{}_{{}}^{177} {\text{Lu}}}} } \right) = a_{{{}_{{}}^{177} {\text{Lu}}}} D_{{{}_{{}}^{177} {\text{Lu}}}}^{2} + b_{{{}_{{}}^{177} {\text{Lu}}}} D_{{{}_{{}}^{177} {\text{Lu}}}}$$

5 $$N_{{{\text{DSB}}, {}_{{}}^{225} {\text{Ac}}}} \left( {D_{{{}_{{}}^{225} {\text{Ac}}}} } \right) = b_{{{}_{{}}^{225} {\text{Ac}}}} D_{{{}_{{}}^{225} {\text{Ac}}}}$$

where $D_{{{}_{{}}^{177} {\text{Lu}}}}$ and $D_{{{}_{{}}^{225} {\text{Ac}}}}$ denote the absorbed dose to the nucleus caused by ^177^Lu and ^225^Ac, respectively. Setting $N_{{{\text{DSB}}, {}_{{}}^{177} {\text{Lu}}}} \left( {D_{{{}_{{}}^{177} {\text{Lu}}}} } \right) = N_{{{\text{DSB}}, {}_{{}}^{225} {\text{Ac}}}} \left( {D_{{{}_{{}}^{225} {\text{Ac}}}} } \right)$ (isoeffect) produces the equation $a_{{{}_{{}}^{177} {\text{Lu}}}} D_{{{}_{{}}^{177} {\text{Lu}}}}^{2} + b_{{{}_{{}}^{177} {\text{Lu}}}} D_{{{}_{{}}^{177} {\text{Lu}}}} = b_{{{}_{{}}^{225} {\text{Ac}}}} D_{{{}_{{}}^{225} {\text{Ac}}}}$, which allows to express $D_{{{}_{{}}^{225} {\text{Ac}}}}$ in terms of $D_{{{}_{{}}^{177} {\text{Lu}}}}$ as:

$$D_{{{}_{{}}^{225} {\text{Ac}}}} = \frac{{a_{{{}_{{}}^{177} {\text{Lu}}}} }}{{b_{{{}_{{}}^{225} {\text{Ac}}}} }}D_{{{}_{{}}^{177} {\text{Lu}}}}^{2} + \frac{{b_{{{}_{{}}^{177} {\text{Lu}}}} }}{{b_{{{}_{{}}^{225} {\text{Ac}}}} }}D_{{{}_{{}}^{177} {\text{Lu}}}} ,$$

or vice versa, $D_{{{}_{{}}^{177} {\text{Lu}}}}$ in terms of $D_{{{}_{{}}^{225} {\text{Ac}}}}$ as:

$$D_{{{}_{{}}^{177} {\text{Lu}}}} = \frac{{\sqrt {b_{{{}_{{}}^{177} {\text{Lu}}}}^{2} + 4 a_{{{}_{{}}^{177} {\text{Lu}}}} b_{{{}_{{}}^{225} {\text{Ac}}}} D_{{{}_{{}}^{225} {\text{Ac}}}} } - b_{{{}_{{}}^{177} {\text{Lu}}}} }}{{2a_{{{}_{{}}^{177} {\text{Lu}}}} }}.$$

Substituting $D_{{{}_{{}}^{225} {\text{Ac}}}} = \frac{{a_{{{}_{{}}^{177} {\text{Lu}}}} }}{{b_{{{}_{{}}^{225} {\text{Ac}}}} }}D_{{{}_{{}}^{177} {\text{Lu}}}}^{2} + \frac{{b_{{{}_{{}}^{177} {\text{Lu}}}} }}{{b_{{{}_{{}}^{225} {\text{Ac}}}} }}D_{{{}_{{}}^{177} {\text{Lu}}}}$ or $D_{{{}_{{}}^{177} {\text{Lu}}}} = \frac{{\sqrt {b_{{{}_{{}}^{177} {\text{Lu}}}}^{2} + 4 a_{{{}_{{}}^{177} {\text{Lu}}}} b_{{{}_{{}}^{225} {\text{Ac}}}} D_{{{}_{{}}^{225} {\text{Ac}}}} } - b_{{{}_{{}}^{177} {\text{Lu}}}} }}{{2a_{{{}_{{}}^{177} {\text{Lu}}}} }}$ in the quotient ${\text{RBE}}_{{{}_{{}}^{225} {\text{Ac}}}} = \left. {\frac{{D_{{{}_{{}}^{177} {\text{Lu}}}} }}{{D_{{{}_{{}}^{225} {\text{Ac}}}} }}} \right|_{{N_{{{\text{DSB}}, {}_{{}}^{177} {\text{Lu }}}} \left( {D_{{{}_{{}}^{177} {\text{Lu}}}} } \right) = N_{{{\text{DSB}}, {}_{{}}^{225} {\text{Ac}}}} \left( {D_{{{}_{{}}^{225} {\text{Ac}}}} } \right) }}$ leads to the RBE of ^225^Ac as a function of $D_{{{}_{{}}^{177} {\text{Lu}}}}$ ${\text{RBE}}_{{{}_{{}}^{225} {\text{Ac}}}} \left( {D_{{{}_{{}}^{177} {\text{Lu}}}} } \right)$ or as a function of $D_{{{}_{{}}^{225} {\text{Ac}}}}$ ${\text{RBE}}_{{{}_{{}}^{225} {\text{Ac}}}} \left( {D_{{{}_{{}}^{225} {\text{Ac}}}} } \right)$, respectively:

6 $${\text{RBE}}_{{{}_{{}}^{225} {\text{Ac}}}} \left( {D_{{{}_{{}}^{177} {\text{Lu}}}} } \right) = \frac{{\frac{{b_{{{}_{{}}^{225} {\text{Ac}}}} }}{{b_{{{}_{{}}^{177} {\text{Lu}}}} }}}}{{\frac{{a_{{{}_{{}}^{177} {\text{Lu}}}} }}{{b_{{{}_{{}}^{177} {\text{Lu}}}} }} D_{{{}_{{}}^{177} {\text{Lu}}}} + 1}}$$

7 $${\text{RBE}}_{{{}_{{}}^{225} {\text{Ac}}}} \left( {D_{{{}_{{}}^{225} {\text{Ac}}}} } \right) = \frac{{2 b_{{{}_{{}}^{225} {\text{Ac}}}} }}{{\sqrt {b_{{{}_{{}}^{177} {\text{Lu}}}}^{2} + 4 a_{{{}_{{}}^{177} {\text{Lu}}}} b_{{{}_{{}}^{225} {\text{Ac}}}} D_{{{}_{{}}^{225} {\text{Ac}}}} } + b_{{{}_{{}}^{177} {\text{Lu}}}} }}$$

Both functions are monotonically decreasing and the following limits are valid:

8 $$\mathop {\lim }\limits_{{D_{{{}_{{}}^{177} {\text{Lu}}}} \to 0}} {\text{RBE}}_{{{}_{{}}^{225} {\text{Ac}}}} \left( {D_{{{}_{{}}^{177} {\text{Lu}}}} } \right) = \mathop {\lim }\limits_{{D_{{{}_{{}}^{225} {\text{Ac}}}} \to 0}} {\text{RBE}}_{{{}_{{}}^{225} {\text{Ac}}}} \left( {D_{{{}_{{}}^{225} {\text{Ac}}}} } \right) = \frac{{b_{{{}_{{}}^{225} {\text{Ac}}}} }}{{b_{{{}_{{}}^{177} {\text{Lu}}}} }}$$

Interestingly, Eq. 6 allows one to determine the RBE of ^225^Ac based on the absorbed dose to the nucleus caused by ^177^Lu, given that the effects produced by ^225^Ac and ^177^Lu are equal. It is worth mentioning that Eqs. 6 and 7 apply to any radiation types if the dose–effect relationship of the investigated radiation type is linear and that of the reference radiation type is linear-quadratic.

Analogous formulas were derived by Hobbs et al. [8] for the relative biological effectiveness in the context of alpha particle radiopharmaceutical therapy based on the surviving fraction of cells receiving an absorbed dose from low-LET radiation or alpha particles. In particular, the authors have shown that this RBE depends on the surviving fraction of cells chosen, or equivalently on the absorbed dose caused by low-LET radiation or alpha particles and no dose-independent resolution for the value of RBE is possible.

## Results

### Visualization

**Fig. 6:**
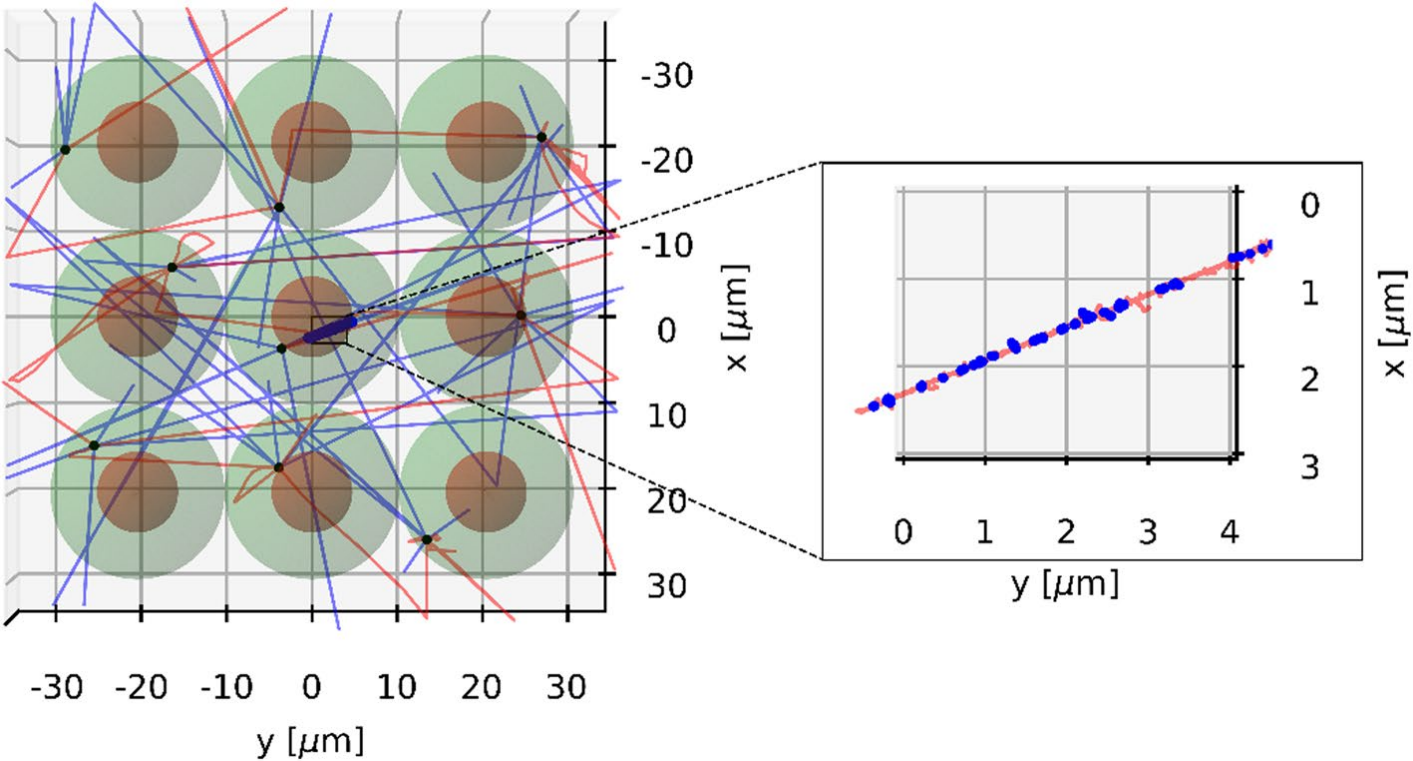
Visualization of an exemplary simulation with ^225^Ac. The number of source points per cell was set to 1. The tracks of alpha particles and electrons are indicated with red and blue lines, respectively. The DSBs are shown as blue dots. The source points are depicted as black dots

**Fig. 7:**
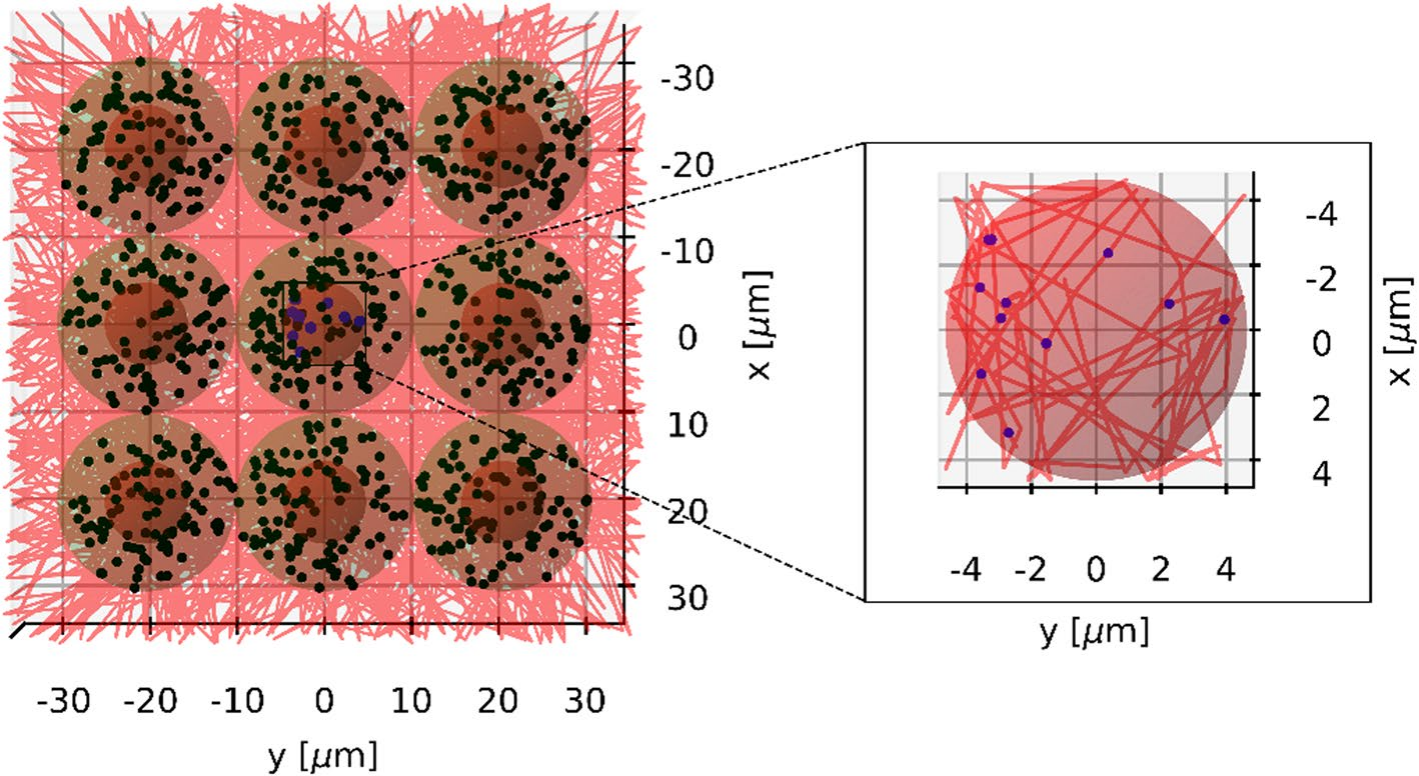
Visualization of an exemplary simulation with ^177^Lu. The number of source points per cell was set to 100. The tracks of electrons are indicated with red lines. The DSBs are shown as blue dots. The source points are depicted as black dots

The simulations were run with graphics turned off, because building the geometry components with visualization switched on would significantly slow down the simulations. Figures 6 and 7 visualize the simulation process for both ^225^Ac and ^177^Lu based on a small cell cluster. As one can see, alpha particles emitted by ^225^Ac that hit the target (nucleus of the central cell) produce a lot of DSBs along their tracks, whereas electrons emitted by ^177^Lu yield DSBs that are sparsely distributed over the entire nucleus.

### Dose–effect relationship

**Fig. 8:**
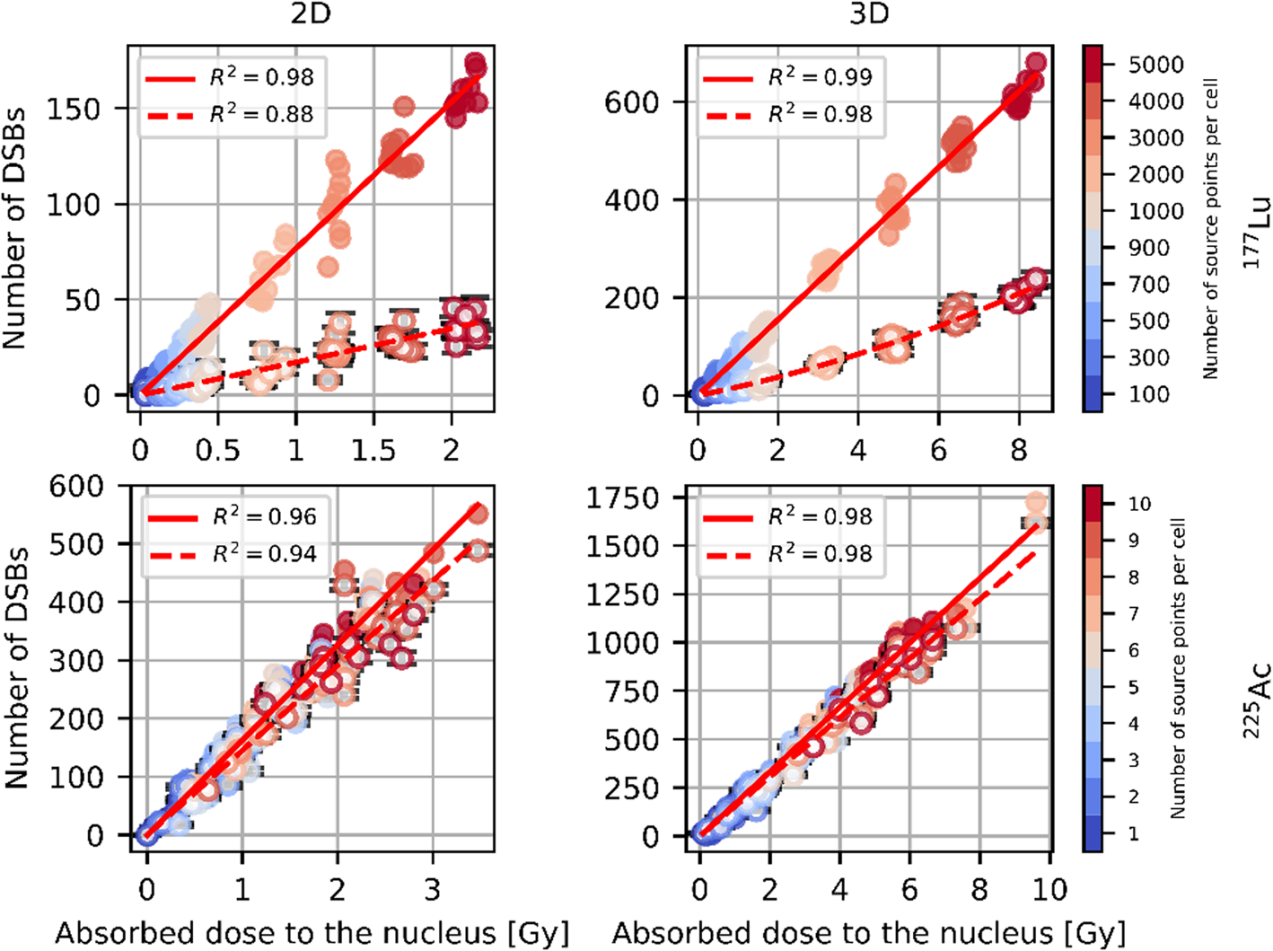
Exemplary dose–effect curves of ^177^Lu and ^225^Ac for cell geometry 1 and full internalization. Initial DSBs are shown as full circles, post-repair DSBs are depicted as circles with white background color. Error bars of number of DSBs after repair are shown in black

**Fig. 9:**
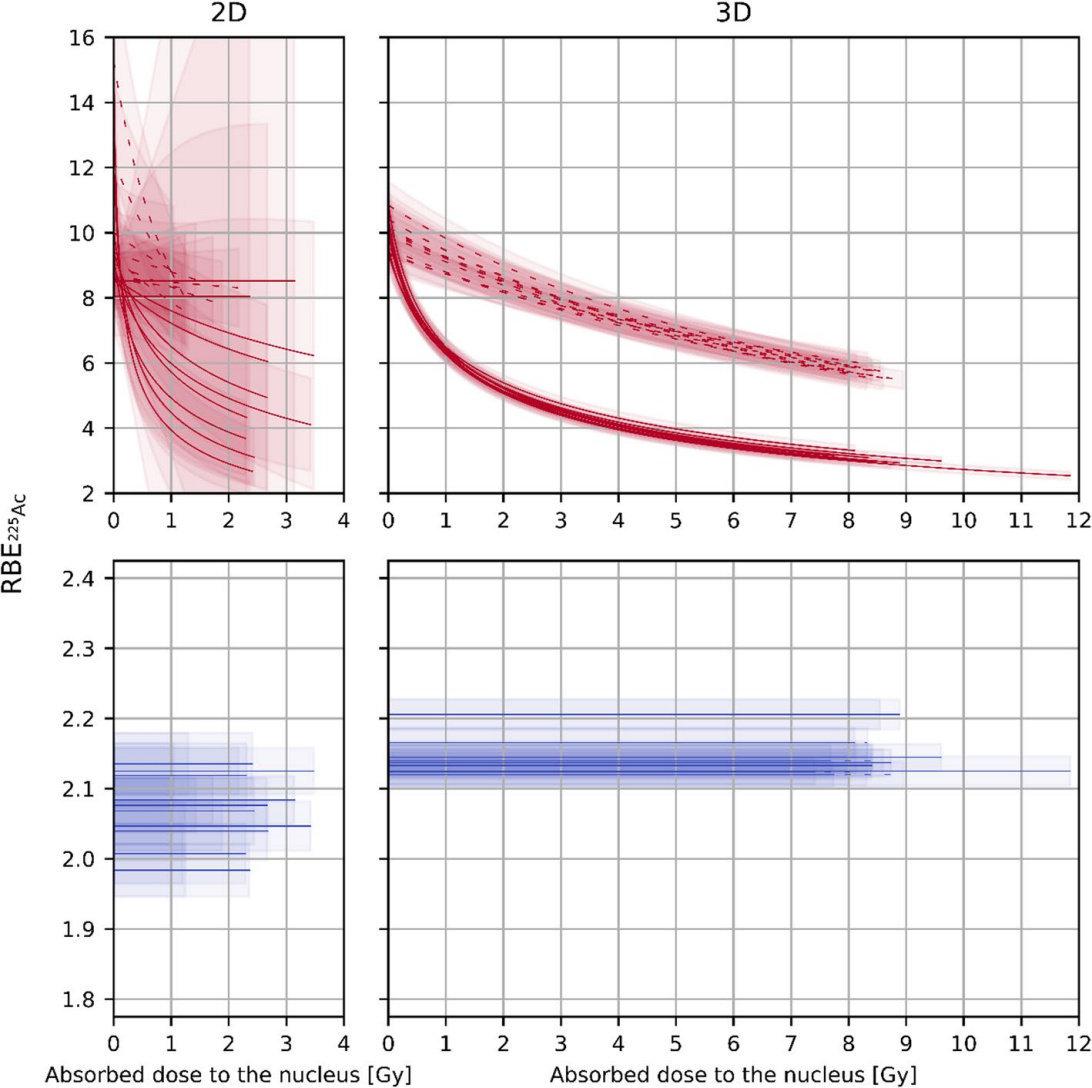
RBE of ^225^Ac as a function of the absorbed dose to the nucleus. Dashed curves represent ${\text{RBE}}_{{{}_{{}}^{225} {\text{Ac}}}} \left( {D_{{{}_{{}}^{177} {\text{Lu}}}} } \right)$. ${\text{RBE}}_{{{}_{{}}^{225} {\text{Ac}}}} \left( {D_{{{}_{{}}^{225} {\text{Ac}}}} } \right)$ is shown with solid curves. RBEs based on the initial damage and the post-repair damage are depicted in blue and red, respectively. The uncertainties are displayed as bands

In Fig. 8, the initial and post-repair dose–effect curves of ^177^Lu and ^225^Ac are shown for cell geometry 1 and full internalization. The remaining dose–effect curves can be found in the Additional file 1. As can be seen from Fig. 8 and from Additional file 1: Figures s1–s4, while $N_{{{\text{DSB}}}}$ is linear with $D$ both for the initial damage and post-repair damage in the case of ^225^Ac, it exhibits a curvature as a function of the absorbed dose to the nucleus after repair in the case of ^177^Lu, which is particularly noticeable for the 3D cell arrangement scenario. For the initial damage caused by ^177^Lu, the linear-quadratic fit yielded $a_{{{}_{{}}^{177} {\text{Lu}}}} = 0$ or very small values of $a_{{{}_{{}}^{177} {\text{Lu}}}}$ in most cases and produced outliers with high $a_{{{}_{{}}^{177} {\text{Lu}}}}$ values in a smal number of cases. Therefore, for the initial damage caused by ^177^Lu, a linear fit was used instead. Using a linear dose–effect relationship for the initial damage for ^177^Lu ensured that there were no outliers, provided smaller uncertainties of $b_{{{}_{{}}^{177} {\text{Lu}}}}$ and $R^{2}$ values very similar to those obtained in the case of the linear-quadratic fit. Curve fitting was performed using a „curve fit “ function provided by the SciPy Python library. The goodness of fit was reported based on the coefficient of determination $R^{2}$. The estimated fit parameters are displayed in Tables 3 and 4. Based on the determined parameters, RBE was plotted as a function of $D_{{{}_{{}}^{177} {\text{Lu}}}}$ and $D_{{{}_{{}}^{225} {\text{Ac}}}}$ for each cell geometry and internalization scenario without and with DNA repair considered separately for the 2D and 3D cell arrangement scenario in the respective simulated absorbed dose range (see Fig. 9). The uncertainties of RBE were calculated using the propagation of uncertainty (see Additional file 1). It should be noted that the Eqs. 6 and 7 and the formulas for the uncertainty of RBE of ^225^Ac in the supplementary information derived using a linear-quadratic dose–effect relationship for ^177^Lu $N_{{{\text{DSB}}, {}_{{}}^{177} {\text{Lu}}}} \left( {D_{{{}_{{}}^{177} {\text{Lu}}}} } \right) = a_{{{}_{{}}^{177} {\text{Lu}}}} D_{{{}_{{}}^{177} {\text{Lu}}}}^{2} + b_{{{}_{{}}^{177} {\text{Lu}}}} D_{{{}_{{}}^{177} {\text{Lu}}}}$ can still be applied to the case of a linear fit by setting $a_{{{}_{{}}^{177} {\text{Lu}}}}$ to zero.

**Table 3:** Estimated fit parameters for ^177^Lu

Cell geom.	Intern.		$b_{{{\text{init}}}} , {\text{DSB}}s \, {\text{Gy}}^{ - 1}$	$b_{{{\text{repair}}}} , {\text{DSB}}s\,{\text{ Gy}}^{ - 1}$	$a_{{{\text{repair}}}} ,{\text{ DSB}}s\,{\text{ Gy}}^{ - 2}$
1	int.	2D	76.75 ± 0.82	16.84 ± 1.57	0.30 ± 0.91
1	membr.	2D	77.17 ± 0.86	17.00 ± 1.68	0.00 ± 1.23
2	int.	2D	79.13 ± 1.02	15.91 ± 1.91	1.36 ± 1.33
2	membr.	2D	78.00 ± 1.13	15.42 ± 2.08	1.06 ± 1.88
3	int.	2D	79.31 ± 1.23	17.13 ± 2.53	0.00 ± 2.58
3	membr.	2D	77.18 ± 1.35	11.63 ± 2.35	4.51 ± 2.95
4	int.	2D	78.28 ± 1.13	14.84 ± 2.01	2.67 ± 2.15
4	membr.	2D	76.40 ± 1.13	9.43 ± 2.08	6.89 ± 2.01
5	int.	2D	78.78 ± 1.11	16.54 ± 2.17	0.43 ± 2.26
5	membr.	2D	75.57 ± 1.03	14.27 ± 2.26	1.87 ± 2.82
1	int.	3D	77.69 ± 0.43	16.31 ± 0.81	1.21 ± 0.12
1	membr.	3D	78.28 ± 0.44	15.50 ± 0.99	1.45 ± 0.16
2	int.	3D	76.89 ± 0.47	15.05 ± 1.10	1.32 ± 0.16
2	membr.	3D	78.93 ± 0.39	14.87 ± 0.88	1.49 ± 0.14
3	int.	3D	76.94 ± 0.44	14.41 ± 0.96	1.49 ± 0.14
3	membr.	3D	76.59 ± 0.43	16.35 ± 0.94	1.11 ± 0.14
4	int.	3D	77.19 ± 0.43	14.48 ± 1.09	1.48 ± 0.16
4	membr.	3D	77.57 ± 0.37	15.73 ± 0.85	1.30 ± 0.12
5	int.	3D	77.41 ± 0.43	14.86 ± 1.09	1.48 ± 0.16
5	membr.	3D	77.20 ± 0.49	15.10 ± 1.10	1.32 ± 0.17

$a_{{{\text{repair}}}}$ denote $a_{{{}_{{}}^{177} {\text{Lu}}}}$ defined in Methods for the post-repair damage

$b_{{{\text{init}}}}$ and $b_{{{\text{repair}}}}$ denote $b_{{{}_{{}}^{177} {\text{Lu}}}}$ described in Methods for the initial damage and the post-repair damage, rescpectively

**Table 4:** Estimated fit parameters for ^225^Ac

Cell geom.	Intern.		$b_{{{\text{init}}}} , {\text{DSB}}s \, {\text{Gy}}^{ - 1}$	$b_{{{\text{repair}}}} , {\text{DSB}}s\, {\text{ Gy}}^{ - 1}$
1	int.	2D	163.10 ± 1.82	145.36 ± 1.90
1	membr.	2D	160.81 ± 1.94	144.95 ± 1.98
2	int.	2D	161.95 ± 1.81	143.88 ± 1.96
2	membr.	2D	161.97 ± 1.91	144.90 ± 2.04
3	int.	2D	157.35 ± 1.74	137.71 ± 1.75
3	membr.	2D	159.63 ± 2.35	142.29 ± 2.68
4	int.	2D	157.15 ± 2.44	137.76 ± 2.59
4	membr.	2D	163.13 ± 2.37	143.89 ± 2.47
5	int.	2D	160.69 ± 2.36	141.67 ± 2.51
5	membr.	2D	160.12 ± 2.73	142.74 ± 2.84
1	int.	3D	166.60 ± 1.16	152.99 ± 1.23
1	membr.	3D	166.96 ± 1.13	154.34 ± 1.20
2	int.	3D	164.56 ± 1.36	150.75 ± 1.46
2	membr.	3D	167.69 ± 1.17	154.83 ± 1.25
3	int.	3D	169.71 ± 1.32	156.25 ± 1.39
3	membr.	3D	165.86 ± 1.30	152.59 ± 1.36
4	int.	3D	164.95 ± 1.14	150.78 ± 1.21
4	membr.	3D	164.47 ± 1.17	149.65 ± 1.20
5	int.	3D	164.49 ± 1.32	150.94 ± 1.40
5	membr.	3D	165.55 ± 1.34	152.14 ± 1.40

$b_{{{\text{init}}}}$ and $b_{{{\text{repair}}}}$ denote $b_{{{}_{{}}^{225} {\text{Ac}}}}$ defined in Methods for the initial damage and the post-repair damage, rescpectively

It can be seen from Fig. 9 that the RBE values calculated on the basis of the 2D data are in agreement with those obtained based on the 3D data, but the former have larger uncertainties. For example, considering only the initial damage, the RBE of ^225^Ac at $0 \;{\text{Gy}}$ varies between 1.984 and 2.135 with an uncertainty ranging between 0.033 and 0.047 and 2.120 and 2.206 with an uncertainty ranging between 0.018 and 0.022 depending on cell geometry and internalization assumption for the 2D and 3D cell arrangement scenario, respectively (please note that ${\text{RBE}}_{{{}_{{}}^{225} {\text{Ac}}}} \left( {D_{{{}_{{}}^{177} {\text{Lu}}}} = 0} \right) = {\text{RBE}}_{{{}_{{}}^{225} {\text{Ac}}}} \left( {D_{{{}_{{}}^{225} {\text{Ac}}}} = 0} \right) = \frac{{b_{{{}_{{}}^{225} {\text{Ac}}}} }}{{b_{{{}_{{}}^{177} {\text{Lu}}}} }}$). For the post-repair damage, in the case of 2D cell arrangement, the RBE of ^225^Ac at $0 \;{\text{Gy}}$ varies between 8.04 and 10.00 with an uncertainty ranging between 0.81 and 1.59 depending on cell geometry and internalization assumption and feautures two outliers—12.23 ± 2.49 and 15.25 ± /3.37—in the case of radionuclide distribution on the cell membrane for cell geometry 3 and 4, respectively. Accordingy, the RBE of ^225^Ac at $0 \;{\text{Gy}}$ varies between 9.33 and 10.84 with an uncertainty ranging between 0.47 and 0.79 depending on cell geometry and internalization assumption in the 3D cell arrangement case. In the 2D case, fewer cells and thus fewer source points were simulated, resulting in smaller absorbed doses to the nucleus and a larger spread of the scored quantities. Comparing the 2D fit parameters with the 3D fit parameters, we find that the relative deviation for the parameter $b_{{{}_{{}}^{177} {\text{Lu}}}}$ without consideration of DNA repair varies between − 3% and 3% depending on cell geometry and internalization scenario. Accordingly, the relative deviation of $b_{{{}_{{}}^{177} {\text{Lu}}}}$ with consideration of DNA repair varies between − 6% and 19% featuring two outliers − 41% and − 29%—in the case of radionuclide distribution on the cell membrane for cell geometry 3 and 4, respectively. These outliers are due to the fact that for the 2D cell arrangement with DNA repair taken into account, the parameter $b_{{{}_{{}}^{177} {\text{Lu}}}}$ in the case of radionuclide distribution on the cell membrane for cell geometry 3 and 4 is significantly smaller than in other cases, resulting in higher relative deviations of $b_{{{}_{{}}^{177} {\text{Lu}}}}$ from the 3D case and higher RBE values of ^225^Ac at $0\;{\text{ Gy}}$ (12.23 ± 2.49 and 15.25 ± 3.37) in the 2D case. The parameter $b_{{{}_{{}}^{225} {\text{Ac}}}}$ is 1% to 8% and 4% to 12% smaller in the 2D case than in the 3D case, without and with DNA repair, respectively. For the parameter $a_{{{}_{{}}^{177} {\text{Lu}}}}$ with consideration of DNA repair, the comparison of the 2D data with the 3D data is not meaningful due to the fact that in most cases, the uncertainty of $a_{{{}_{{}}^{177} {\text{Lu}}}}$ is greater than the estimated value for the 2D cell arrangement scenario.

If only the initial damage is considered, the RBE of ^225^Ac is independent of the absorbed dose to the nucleus and can be expressed as

9 $${\text{RBE}}_{{{}_{{}}^{225} {\text{Ac}}}} \left( {D_{{{}_{{}}^{177} {\text{Lu}}}} } \right) = {\text{RBE}}_{{{}_{{}}^{225} {\text{Ac}}}} \left( {D_{{{}_{{}}^{225} {\text{Ac}}}} } \right) = \frac{{b_{{{}_{{}}^{225} {\text{Ac}}}} }}{{b_{{{}_{{}}^{177} {\text{Lu}}}} }}\;{\text{ for}}\; D_{{{}_{{}}^{177} {\text{Lu}}}} , D_{{{}_{{}}^{225} {\text{Ac}}}} \ge 0$$

by setting $a_{{{}_{{}}^{177} {\text{Lu}}}} = 0$ in Eqs. 6 and 7.

As can be seen in Fig. 9, in the case of 3D cell arrangement with DNA repair considered, the function ${\text{RBE}}_{{{}_{{}}^{225} {\text{Ac}}}} \left( {D_{{{}_{{}}^{225} {\text{Ac}}}} } \right)$ drops faster than ${\text{RBE}}_{{{}_{{}}^{225} {\text{Ac}}}} \left( {D_{{{}_{{}}^{177} {\text{Lu}}}} } \right)$ in the plotted absorbed dose range for each cell geometry and internalization scenario. For each cell geometry and internalization scenario, the RBE as a function of the absorbed dose to the nucleus caused by ^225^Ac is smaller than the RBE as a function of the absorbed dose to the nucleus caused by ^177^Lu for absorbed doses to the nucleus $0 < D < \frac{{b_{{{}_{{}}^{225} {\text{Ac}}}} - b_{{{}_{{}}^{177} {\text{Lu}}}} }}{{a_{{{}_{{}}^{177} {\text{Lu}}}} }}$, ${\text{RBE}}_{{{}_{{}}^{225} {\text{Ac}}}} \left( {D_{{{}_{{}}^{177} {\text{Lu}}}} } \right) = {\text{ RBE}}_{{{}_{{}}^{225} {\text{Ac}}}} \left( {D_{{{}_{{}}^{225} {\text{Ac}}}} } \right) = 1$ is valid at $D = \frac{{b_{{{}_{{}}^{225} {\text{Ac}}}} - b_{{{}_{{}}^{177} {\text{Lu}}}} }}{{a_{{{}_{{}}^{177} {\text{Lu}}}} }}$ and for $D > \frac{{b_{{{}_{{}}^{225} {\text{Ac}}}} - b_{{{}_{{}}^{177} {\text{Lu}}}} }}{{a_{{{}_{{}}^{177} {\text{Lu}}}} }}$, ${\text{RBE}}_{{{}_{{}}^{225} {\text{Ac}}}} \left( {D_{{{}_{{}}^{225} {\text{Ac}}}} } \right)$ is greater than ${\text{RBE}}_{{{}_{{}}^{225} {\text{Ac}}}} \left( {D_{{{}_{{}}^{177} {\text{Lu}}}} } \right)$ (see the mathematical derivation in the Additional file 1). An example of overlaying ${\text{RBE}}_{{{}_{{}}^{225} {\text{Ac}}}} \left( {D_{{{}_{{}}^{225} {\text{Ac}}}} } \right)$ and ${\text{RBE}}_{{{}_{{}}^{225} {\text{Ac}}}} \left( {D_{{{}_{{}}^{177} {\text{Lu}}}} } \right)$ for the 3D cell arrangement, cell geometry 1 and full internalization with DNA repair in a large absorbed dose interval can be found in Additional file 1: Figure s12. As can be seen from this example, in the case of 3D cell arrangement, cell geometry 1 and full internalization with DNA repair considered, the absorbed dose at which $N_{{{\text{DSB}}, {}_{{}}^{225} {\text{Ac}}}}$ is equal to $N_{{{\text{DSB}}, {}_{{}}^{177} {\text{Lu}}}}$ and thus ${\text{RBE}}_{{{}_{{}}^{{{225}}} {\text{Ac}}}} \left( {D_{{{}_{{}}^{225} {\text{Ac}}}} } \right)$ equals to ${\text{RBE}}_{{{}_{{}}^{225} {\text{Ac}}}} \left( {D_{{{}_{{}}^{177} {\text{Lu}}}} } \right)$ is 113 Gy ± 12 Gy. It should be noted that the maximum absorbed dose to the nucleus obtained from the simulations was about 12 Gy (^225^Ac, 3D, cell geometry 1, full internalization), which is much smaller than the calculated value. At very high absorbed doses to the nucleus, the dose dependence of the number of DSBs might contain a quadratic term for the high-LET radiation.

For the initial DNA damage induced by ^177^Lu in the 3D cell arrangement case, the DSB yield in terms of DSBs normalized to the absorbed dose to the nucleus in Gy and the number of base pairs in Gbp ranged between (12.60 ± 0.26) DSBs Gy^−1^ Gbp^−1^ and (12.78 ± 0.26) DSBs Gy^−1^ Gbp^−1^ in the cases of a linear dose–effect relationship ($a_{{{}_{{}}^{177} {\text{Lu}}}} = 0 {\text{ DSBs}} {\text{ Gy}}^{ - 2}$; see Table 3), depending on the cell geometry and internalization scenario.

**Fig. 10:**
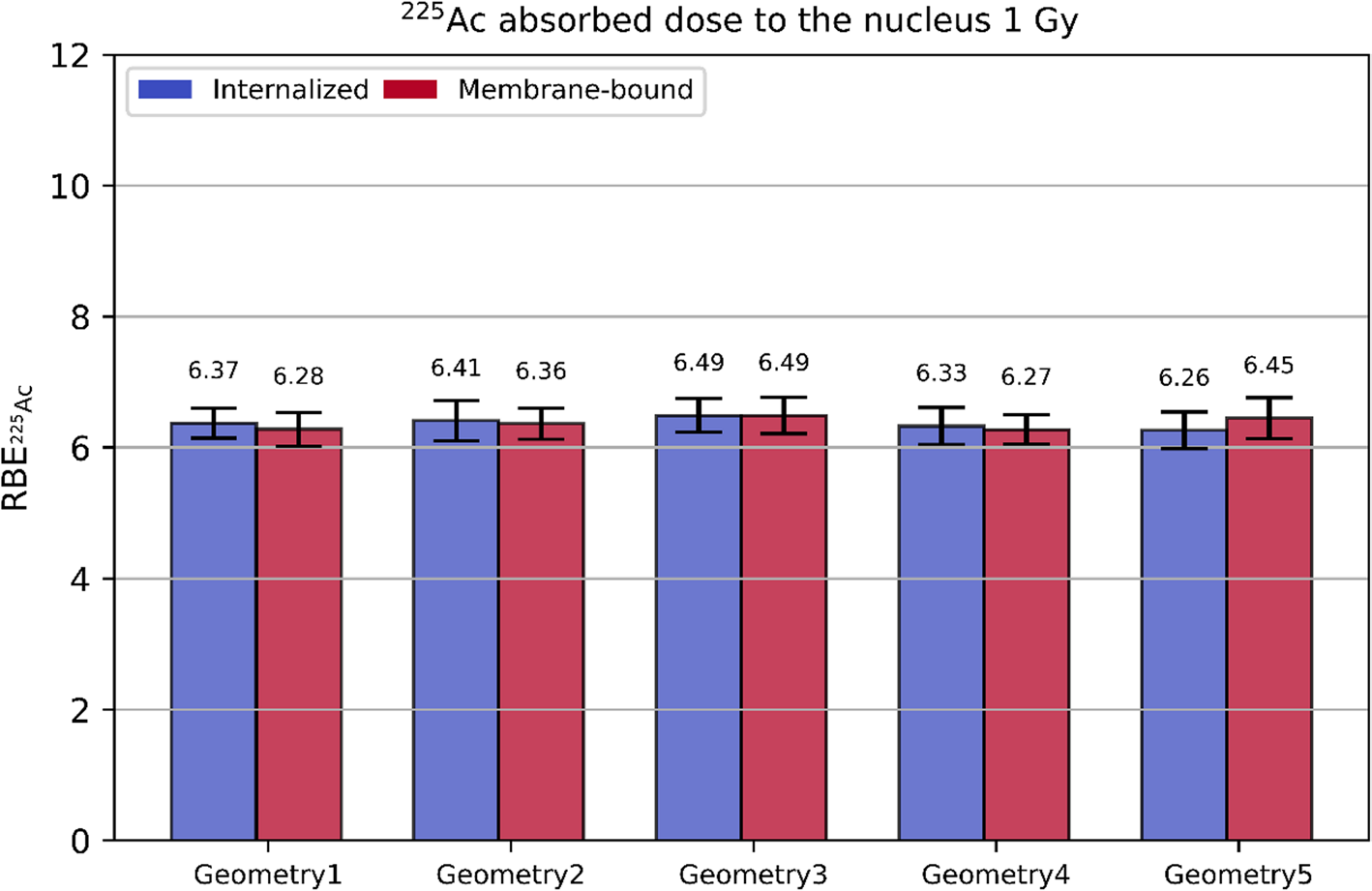
RBE of ^225^Ac at $D_{{{}_{{}}^{225} {\text{Ac}}}} = 1\;{\text{ Gy}}$ based on the 3D data

In Fig. 10, the RBE of ^225^Ac at a reference absorbed dose to the nucleus of 1 Gy caused by ^225^Ac is shown for each cell geometry and radionuclide internalization scenario based on the simulations in the 3D cell arrangement case. As can be seen from Fig. 10, the RBE varies among the considered cell geometries and internalization scenarios, but the values agree with each other within the estimated uncertainties. The RBE of ^225^Ac at different reference absorbed doses to the nucleus of 1 Gy caused by ^225^Ac can be found in the Additional file 1.

## Discussion

In MIRD pamphlet no. 27, Katugampola et al. [50] has presented MIRDcell V3, a revised software tool for multicellular dosimetry and bioeffect modeling in the context of radiopharmaceutical therapy. It combines analytical and Monte Carlo methods to perform dosimetry and bioeffect modeling for radiolabeled cells within 2D and 3D cell populations. This shows that microdosimetry for radionuclide therapy is of great importance and is an ongoing research topic.

The observed linear relationship of the simulated dose–effect curves of ^225^Ac and linear-quadratic relationship for ^177^Lu (for the post-repair damage) are in accordance with a report of the International Commission on Radiological Protection on RBE [51]. According to this report, at low to intermediate absorbed doses, high- and low-LET radiations manifest linear and linear-quadratic dose–effect curves, respectively. $\alpha$-emitting ^225^Ac presents a high-LET radiation, whereas $\beta^{ - }$-emitting ^177^Lu is considered as a low-LET radiation. Similar DSB yields (14 DSBs Gy^−1^ and 17 DSBs Gy^−1^ per cell with a nucleus containing 6 Gbp) have been reported by Tamborino et al. [46] for the early radiation induced DNA damage occurring during ^177^Lu-DOTATATE therapy, which presents a validation of our approach.

One would expect the RBE to depend on the spatial distribution of the source points and thus on the cell geometry and the internalization and cell arrangement scenario. This can be explained by the fact that the energy of electrons and alpha particles decreases along the track and thus the LET of the particle, which describes the energy loss of a charged particle due to electromagnetic interactions along a track, increases, which leads to a higher induction of DSBs. That means that particles coming from a greater distance and hitting the nucleus have a smaller energy and thus a higher LET and a higher efficiency to induce DSBs. The geometry of the cell and the internalization scenario influence the energy spectrum of particles hitting the cell nucleus. The very low-energy Auger and internal conversion electrons with the highest LET emitted during the decay of ^177^Lu may not reach the nucleus unless they are emitted in the immediate vicinity of the cell nucleus. For example, electrons with an energy of 10 keV have a CSDA range of approx. 2.5 µm according to [45]. The energies of Auger and internal conversion electrons emitted during the decay of ^177^Lu can be found, for example, in [52] and [53]. In the case of ^225^Ac, alpha particles with the lowest energy ($E_{\alpha } = 5.8 \;{\text{MeV}}$) have a CSDA range between 43 µm and 49 µm (see [45]). If the ^225^Ac source points were distributed on the cell membrane with a distance from the cell nucleus greater than 50 µm, these alpha particles would have a low probability to reach the nucleus. In our simulations, the effects described above were not observed. Firstly, it might be due the fact that the fraction of DSBs induced by Auger electrons was negligible even in the case of full internalization either because of a small number of Auger electrons being emitted or because of long distances from the nucleus to the sites where the Auger electrons were emitted. It might be also due to fact that the differences in the energy spectra of electrons hitting the nucleus were small among the cell geometries considered. The latter fact has been reported in a study performed by Tamborino et al. [46], according to which the energy distribution of electrons entering the cell nucleus from the surrounding medium is not shifted to lower energies compared to the electrons that originate from ^177^Lu source points distributed within the cell. Secondly, this may be addressed to the dimensions of the cells considered being smaller than the ranges of alpha particles emitted during the decay of ^225^Ac. To increase the accuracy of the RBE estimation, improved descriptions of the cell geometry are desired. This may be achievable by implementing tissue-specific cell geometries and cell arrangements.

For ^177^Lu, in the 3D cell arrangement case, the number of cells was set based on the half CSDA range at the average electron energy (140 µm) instead of the full (280 µm) as done in the study by Tamborino et al. [46] to reduce the number of geometry components to be built and thus the computational cost. This might lead to an underestimation of the number of electrons entering the nucleus of the central cell and thus of the number of induced DSBs and the absorbed dose to the nucleus. To show that reducing the cell cluster size by a factor of 2 in each dimension results in a negligible underestimation of the number of DSBs and the absorbed dose to the nucleus, a 2D cell cluster consisting of 15 × 15 = 225 cells (cell geometry 1, full internalization, 5000 source points per cell) was simulated and the simulation results were compared with those obtained from the simulation of a 29 × 29 cluster used in this work. The mean absorbed dose to the nucleus of 10 histories was 2.027 Gy ± 0.097 Gy and 2.084 Gy ± 0.060 Gy, for the 15 × 15 and 29 × 29 cell cluster, respectively, while the mean initial number of DSBs and the mean number of DSBs after DNA repair were estimated as 155.9 DSBs ± 17.8 DSBs and 36.9 DSBs ± 3.2 DSBs and as 157.3 DSBs ± 9.2 DSBs and 37.6 DSBs ± 2.7 DSBs, for the 15 × 15 and 29 × 29 cell cluster, respectively, indicating the largest relative deviation between the corresponding values not exceeding 3%. Decreasing the number of cells surrounding the central cell does not change the dose–effect curve, but only results in a different absorbed dose interval in which this dose–effect curve is simulated, as long as the energy spectrum of electrons entering the nucleus of the central cell is not significantly shifted to lower energies, which leads to a higher LET of the electrons and thus a higher induction of DSBs. As reported by Tamborino et al. [46], the described energy shift is negligible.

To the best of our knowledge, this is the first simulation study estimating the RBE of ^225^Ac compared to ^177^Lu during [^225^Ac]Ac-PSMA and [^177^Lu]Lu-PSMA therapy. A direct comparison of the estimated RBE values with those that can be found in literature is only possible to a limited extent, since there is a lack of experimental data on induction of DSBs by irradiation of cells with ^225^Ac compared to ^177^Lu. Furthermore, the RBE is strongly dependent on the considered biological endpoint and the reference radiation type. Ruigrok et al. [54] investigated the therapeutic efficacy of [^225^Ac]Ac-PSMA-I&T compared to [^177^Lu]Lu-PSMA-I&T by assessing the number of 53BP1 foci (a marker for DSBs) and performing clonogenic cell survival assays. The authors estimated the RBE of [^225^Ac]Ac-PSMA-I&T compared to [^177^Lu]Lu-PSMA-I&T to be 4.2 ± 0.3 based on the cell survival curves. In the ICRU report No. 96 [55], an RBE value of 2.2 and 2.8 was reported for alpha-emitting ^227^Th compared to ^177^Lu using tumor growth delay as a biological endpoint. Based on cell survival curves for breast cancer cells, Rajon et al. [56] derived an RBE value of 3 for alpha particles with an average energy of 2.9 MeV compared to 662 keV gamma rays at an absorbed dose caused by alpha particles of 0.73 Gy. Bastiaannet et al. [57] reported dose-dependent RBE values for alpha-emitter radiopharmaceutical therapy with ^212^Pb, which decays to alpha-emitting ^212^Bi, ranging between 9 and 17.5 derived from surviving fractions of the HER2 + breast cancer cell line treated with a ^212^Pb-labeled anti-HER2 conjugate or external beam radiotherapy (EBRT). Employing EBRT as the reference radiation, Liatsou et al. [58] determined the in vivo RBE of ^212^Pb-labeled anti-HER2/neu antibody in mice using femur marrow cellularity as the biological endpoint and found the dose–response for EBRT and ^212^Pb-anti-HER2/neu antibody to be linear-quadratic and linear, respectively, implying dose-dependent RBE values. On transforming the EBRT dose–response elationship into a linear relationship using the equivalent dose in 2-Gy fractions of EBRT formalism, the authors obtained a dose-independent RBE of 6.4. According to MIRD pamphlet No. 21 [59], for alpha particle emitters, RBE values range from 1 to 8 for cell killing in vivo depending on the reference radiation and alpha particle energy. Although a direct comparison of our RBE values with those being determined for cell killing is not possible, our RBE values seem plausible, at least in terms of order of magnitude.

The values of the initial number of DSBs we obtained depend on the following parameters used in our TOPAS simulations: the threshold for direct damage to the backbone (17.5 eV), the probability for a hydroxil to cause damage when interacting with the backbone (0.4), the DSB separation length (10 bp) and the chemical stage end time (1 ns). These values are the recommended values provided in the documentation of TOPAS-nBio and were used in other studies [36, 41, 42, 60, 61]. The number of DSBs after repair, estimated as the sum of residual and misrepaired breaks, is influenced by the interaction rates of free DSB ends ($\lambda_{f} = 2.07\; {\text{h}}^{ - 1}$, $\lambda_{s} = 0.259\;{\text{ h}}^{ - 1}$) and the repair time (24 h) implemented in MEDRAS [62–64]. Furthermore, It should be mentioned that the nucleus used in this work presents a model of a fibroblast nucleus in the G_0_/G_1_ cell cycle phase. In contrast, in in vivo and in vitro experiments as well as during clinical therapy, the distribution of cell cycle phases of individual cells exposed to irradiation is inhomogeneous. Cells in the different cell cycle phases are differently sensitive to DNA damages due to, among other things, different efficacy of the repair processes during the different phases. In contrast, cells during mitosis or the G_2_ phase present with the highest sensitivity, cells in the G_0_ or late S phase with the highest resistance. Highly proliferating tissues, such as tumors and the haematopoietic system, in which cells are in the active cell cycle have more cells in the sensitive phases than less proliferating tissues. Furthermore, it should be noted that the induction of DSBs in the case of ^177^Lu and ^225^Ac extends over weeks due to their half-lives. Thus, the DNA repair takes place in parallel with the induction of DSBs, in contrast to our simulations in which the induction of DSBs and their repair were simulated sequentially. This can result in a smaller number of DSBs, especially in the case of ^177^Lu. To improve our simulation approach, the modeling of a representative cell population with a distribution of cell cycle phases and the consideration of the effect of low dose rate in terms of simultaneous occuring of induction of DSBs and DNA repair are desired.

It should be mentioned that the simulated decay sites of the daughter nuclides of ^225^Ac might be different from those in the case of real therapy. According to De Kruijff et al. [65], after the alpha decay of ^225^Ac, the daughter nuclides have a recoil energy that is sufficient for the daughter nuclides to detach from the targeting agent molecule. Considering the membrane-bound ^225^Ac, the resulting free radionuclides not bound to the targeting agent molecule can be transported away from the tumor cell by diffusion and/or blood flow. Thus, the energy deposition in the cell nucleus may change. While ^221^Fr and ^217^At have very short half-lives and thus decay predominantly locally, the decay site of ^213^ Bi, which has a half-life of 45.6 min, may be significantly different from the original decay site of ^225^Ac. If ^225^Ac is internalized, the daughter nuclides will most likely remain in the cell. However, even in this case, ^213^Bi might diffuse into the extracellular space and be transported away due to its comparatively long half-life. These effects were not considered in the simulations. The radioactive daugther nuclides are tracked to zero energy and then decay. For example, if ^225^Ac is at rest, the kinetic energies of ^221^Fr and ^217^At are in the sub-MeV range and the track lengths of ^221^Fr and ^217^At are in the nanometre range, so that all alpha decays occur in a very small vicinity of the initial site of ^225^Ac (see Fig. 6).

**Fig. 11:**
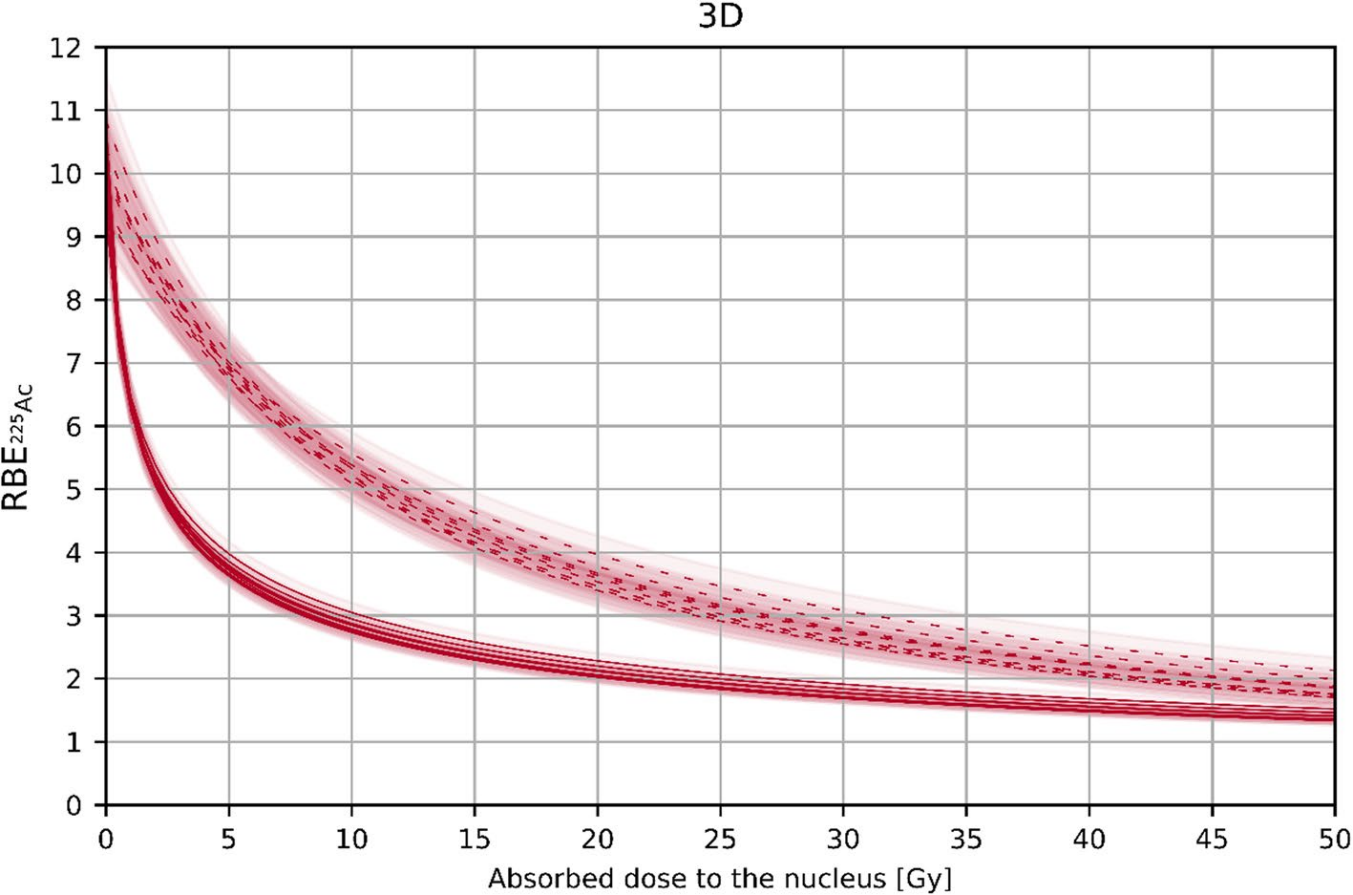
RBE of ^225^Ac as a function of the absorbed dose to the nucleus based on the post-repair damage. Dashed curves represent ${\text{RBE}}_{{{}_{{}}^{225} {\text{Ac}}}} \left( {D_{{{}_{{}}^{177} {\text{Lu}}}} } \right)$. ${\text{RBE}}_{{{}_{{}}^{225} {\text{Ac}}}} \left( {D_{{{}_{{}}^{225} {\text{Ac}}}} } \right)$ is shown with solid curves

Nevertheless, the obtained findings can have implications in clinical dosimetry. Here, we focus on the RBE values obtained for the 3D cell arrangement case, since these have lower uncertainties and the 3D cell arrangement scenarios are more suitable for the description of a real therapy. Extrapolating the RBE as a function of the ^225^Ac absorbed dose to the nucleus up to 50 Gy (see Fig. 11), the corresponding RBE value can be assigned to the absorbed dose caused by [^225^Ac]Ac-PSMA-I&T or [^225^Ac]Ac-PSMA-617 therapy. To allow for reporting on RBE-weighted absorbed dose (in Sv), the estimated absorbed energy dose (in Gy, using the MIRD scheme based on the activities from SPECT images) could be scaled by the associated tissue-specific RBE value from the derived ${\text{RBE}}_{{{}_{{}}^{225} {\text{Ac}}}} \left( {D_{{{}_{{}}^{225} {\text{Ac}}}} } \right)$ parametrization. This implies the assumption that the mean absorbed dose to the nucleus can be inferred from or well approximated by the mean absorbed dose to the whole tissue (organ or lesion). The fact that the RBE value varies with the total absorbed dose and thus amount of injected activity should be kept in mind when RBE-weighted absorbed doses per administered activity are reported.

Another potential application of our simulation results arises from the dependence of the RBE of ^225^Ac on the absorbed dose to the nucleus caused by ^177^Lu. If the absorbed dose in the case of [^177^Lu]Lu-PSMA therapy $D_{{{}_{{}}^{177} {\text{Lu}}}}$ is known, the function ${\text{RBE}}_{{{}_{{}}^{225} {\text{Ac}}}} \left( {D_{{{}_{{}}^{177} {\text{Lu}}}} } \right)$ and the definition of RBE $\text{RBE}_{{{}_{{}}^{225} \text{Ac}}} = \left. {\frac{{D_{{{}_{{}}^{177} \text{Lu}}} }}{{D_{{{}_{{}}^{225} \text{Ac}}} }}} \right|_{\text{isoeffect }}$ could be used to determine the absorbed dose for the case of [^225^Ac]Ac-PSMA therapy $D_{{{}_{{}}^{225} {\text{Ac}}}}$ by dividing $D_{{{}_{{}}^{177} {\text{Lu}}}}$ by ${\text{RBE}}_{{{}_{{}}^{225} {\text{Ac}}}} \left( {D_{{{}_{{}}^{177} {\text{Lu}}}} } \right)$, which would result assuming the same biological effect. Then, using empirical data, one could estimate the activity of ^225^Ac to be injected that would be required to achieve the same biological effect. It should be noted that for such an estimation, it must be assumed that the mean organ absorbed dose can be approximated by the absorbed dose to the nucleus and the simulated biological endpoint—the number of DSBs—can be related to a clinical therapy effect.

In the future, our simulations results need to be validated against experimental data by measuring the number of DSBs induced by irradiation with ^177^Lu and ^225^Ac in in vitro cell experiments or mouse models.

## Conclusion

In this work, we simulated the induction of DSBs in cells of various geometries irradiated with ^177^Lu and ^225^Ac and the subsequent DNA repair in a simulation setup which was designed to mimic the situation in PSMA therapy. Based on the dose–effect curves, RBE values of ^225^Ac compared to ^177^Lu were then determined. The dose-dependent RBE values were specified in a wide range of absorbed doses. Thus, these values can improve clinical dosimetry for radionuclide therapy with ^225^Ac and lead to a better understanding of the therapy effect.

### Supplementary Information

**Additional file 1.** Supplementary information mentioned in the manuscript.

## Data Availability

Please contact the corresponding author.
