# Supplementary material for: Estimation of relative biological effectiveness of 225Ac compared to 177Lu during [225Ac]Ac-PSMA and [177Lu]Lu-PSMA radiopharmaceutical therapy using TOPAS/TOPAS-nBio/MEDRAS
Source: EJNMMI Phys. 2023 Sep 11;10:53. doi: 10.1186/s40658-023-00567-2 (PMC10495309; doi:10.1186/s40658-023-00567-2)
Supplement: Supplementary file 1 — Additional file 1. Supplementary information mentioned in the manuscript. [file 40658_2023_567_MOESM1_ESM.docx]

*Supplementary information*

**Physics modules**

In the following, the physics and chemistry modules required for the simulations will be described. The list of physics modules for ^177^Lu contained “g4decay” and “g4radioactivedecay”, which are based on the Geant4 classes G4DecayPhysics and G4RadioactiveDecayPhysics, in order to simulate the radioactive decay of ^177^Lu. Inside the sensitive volume (i.e. cell nucleus) in which the DNA damage scoring was performed, “g4em-dna” was used as an electromagnetic module, as in the work of [66]. The corresponding Geant4 constructor is G4EmDNAPhysics (default constructor). It simulates the very low-energy interactions of particles (electrons, protons, hydrogen atoms, helium atoms ionized once or twice, neutral helium atoms and gammas) with liquid water as a main component of biological medium. For electrons, this constructor includes elastic scattering (G4DNAElastic process class), electronic excitation (G4DNAExcitation), ionisation (G4DNAIonisation), vibrational excitation (G4DNAVibExcitation) and attachment (G4DNAAttachment). These processes are described by the G4DNAChampionElasticModel in the energy range 7.4 eV to 1 MeV, G4DNABornExcitationModel from 9 eV to 1 MeV, G4DNABornIonisationModel from 11 eV to 1 MeV, G4DNASancheExcitationModel from 2 eV to 100 eV and G4DNAMeltonAttachmentModel from 4 eV to 13 eV model classes, respectively. When the chemistry simulation is activated, electrons are tracked till thermalization (meV) and are considered as solvated [28]. Outside the sensitive volume, “g4em-dna” was switched to “g4em-standard opt4”, since high accuracy of particle tracking is not required beyond the boundary of the nucleus. The latter electromagnetic constructor sets the production cut for secondaries (electrons, gammas) to 50 µm. The same physics modules were used for simulations with ^225^Ac. For alpha particles, the G4EmDNAPhysics constructor incorporates nuclear scattering (G4DNAElastic process class) described by the G4DNAIonElasticModel in the range 100 eV to 1 MeV, electronic excitation (G4DNAExcitation) with the G4DNAMillerGreenExcitationModel from 1 keV to 400 MeV, ionisation (G4DNAIonisation) with the G4DNARuddIonisationModel from 0 eV to 400 MeV and the G4DNADingfelderChargeDecreaseModel from 1 keV to 400 MeV for electron capture (G4DNAChargeDecrease). The modules “g4h-phy QGSP BIC HP”, “g4ion-binarycascade”, “g4h-elastic HP” and “g4stopping” required for simulating the hadronic interactions of alpha particles with atomic nuclei were not included because for the considered alpha particles energies (< 10 MeV) the electromagnetic interaction is the primary interaction with a biological medium. In a separate simulation, 500000 alpha particles with an energy of 8.4 MeV, which is the maximum alpha particle energy during the decay chain of ^225^Ac, were simulated in water with taking into account the hadronic interactions. As a result, less than 0.1 % of alpha particles underwent the hadronic interactions (inelastic or elastic).

**Chemistry module**

To simulate water radiolysis, diffusion of radiolytic products and interactions of chemical species, “TsEmDNAChemistry” was utilized. It inherits the dissociation schemes, branching ratios, number of reactions and reaction rates from Geant-DNA (G4EmDNAChemistry class). The set of chemical parameters to be used with “TsEmDNAChemistry” is stored in the “TOPASChemistry.txt” file. It contains the diffusion coefficients at 25 °C of the following species: e^–^_aq_ (solvated electron), ^•^OH (hydroxyl), H^•^ (hydrogen), H_3_O^+^ (hydronium), H_2_ (dihydrogen), OH^–^ (hydroxide) and H_2_O_2_ (hydrogen peroxide). Apart from that, it includes the products of binary reactions between the above mentioned species and the reaction rates (e.g. e^–^_aq_ + e^–^_aq_ → OH^–^ + OH^–^ + H_2_ with a reaction rate of $0.647 \times{10}^{10} L {mol}^{-1} s^{-1}$). The diffusion and reactions of chemical species were modeled using the explicit step-by-step method [67]. The transport of chemical species lasted up to 1 ns with a time step resolution of 0.5 ps.

**Nucleus model**

The “TsNucleus” cell nucleus model provided by TOPAS-nBio was used in this work. It represents a sphere with a radius of 4.65 µm uniformly filled with DNA. This model utilizes the DNA geometry proposed by Bernal et al. [68] and described by Henthorn et al. [69]. In brief, each strand of the DNA double helix consists of nucleotides with a thickness of 0.34 nm that are formed with a half-cylindrical base with a radius of 0.5 nm and a quarter-cylindrical sugar-phosphate group with an outer radius of 1.15 nm. The spherical nucleus is represented by 14328 voxels that are filled with chromatin fibers following a 3D Hilbert space-filling curve. In total, the nucleus contains 46 chromosomes and 6.0779356 Gbp, giving a DNA density of 14.4316 Mbp µm^−3^. An illustration of the nuclear DNA model can be found in [36]. The materials for bases, sugar-phosphate groups and hydration shells are cloned from “G4_WATER” with a density of 1 g cm^−3^, whereas the histone material is copied from “G4_WATER” with a density of 1.407 g cm^−3^.

**Damage scoring**

The “NucleusDNADamage” was used as a scorer for damage produced in the nucleus. A single-strand break (SSB) is considered when the total energy deposited in a backbone exceeds 17.5 eV (direct damage) or a hydroxil interacts with a backbone with a probability of inducing damage of 40 % (indirect damage), or an ionization is produced in a hydration shell and translated into a backbone break (direct damage). These are the default values provided by TOPAS-nBio. A double-strand break (DSB) is defined as exactly two SSBs in complementary strands that are separated by less than 10 bp. A DSB can be direct, indirect or hybrid depending on the type of the SSBs. Base damages are also considered using the same energy threshold for a direct damage to a base and the same probability to induce an indirect damage to a base. Furthermore, histones are used as scavengers for chemical species. The output of the scorer is produced in the SDD (Standard for DNA Damage) format, which is described in [70].

**DNA repair mechanism**

The MEDRAS (Mechanistic DNA Repair And Survival) model described in [62, 63] was used to simulate DNA damage repair. It is a Python script, which takes DNA damage distributions stored in the SDD format as input and probabilistically simulates their repair by modelling the stochastic rejoining of free DNA ends. It includes three mechanisms to repair DSBs: Homologous Recombination (HR), Nonhomologous End Joining (NHEJ) and Microhomology Mediated End Joining (MMEJ). A detailed description of the three processes can be found in [71]. Briefly, in HR, a homologous sister chromatid is used as a template for the correct base sequence of the DNA. It is a repair process with the highest fidelity, which takes place during the S phase of the cell cycle after DNA replication or the G_2_ phase. In NHEJ, the two DNA fragments are directly joined by a DNA ligase if the ends are not spatially separated. It is the most common repair mechanism of DSBs in human cells and it is available throughout the whole cell cycle. Normally, NHEJ introduces DNA alterations, since some bases are lost during repair. MMEJ is the slowest and the most error-prone pathway. It requires the existence of microhomologous DNA sequences and is associated with a deletion. In MEDRAS, the rejoining rate $\zeta$ of free DSB ends is given by $\zeta(d) \propto exp (-\frac{d^{2}}{{2\sigma}^{2}} ),$ where $d$ is the separation between the two ends and $\sigma$ is a characteristic rejoining range [62]. In this model, the DSBs are repaired with exponential kinetics according to a three-phase behaviour: fast kinetics (simple DSBs, via NHEJ), slow kinetics (complex DSBs, via HR or NHEJ) or very slow kinetics (simple and complex DSBs, via MMEJ). The total number of DSBs $N(t)$ as a function of time is described as $N(t) = N_{0}[p_{f} exp (-\lambda_{f}t) + p_{s} exp (-\lambda_{s}t) + p_{m} exp (-\lambda_{m}t)]$,

where each phase is characterized by a repair rate constant $\lambda$ and a probability $p$ of a DSB to be repaired by the corresponding process. The following values were used in the Python implementation of the model: $\frac{\sigma}{R_{nucleus}}=0.04187$ with $R_{nucleus}$ being the nuclear radius, $\lambda_{f} = 2.07 h^{-1}$, $\lambda_{s}= 0.259 h^{-1}$. The repair simulation time limit was set to 24 h. These values are the default values provided by MEDRAS. At this time point, the simulation provided the number of residual breaks, the number of misrepaired breaks and a set values characterizing chromosomal aberrations (e.g. number of acentric chromosomes).

**Computing**

Most of the simulations were performed in a virtual machine created in the Compute Cloud of the Leibniz Supercomputing Centre of the Bavarian Academy of Sciences and Humanities (Garching/Munich, Germany). A flavor consisting of 1/4 of the “hugemem” node with 48 vCPUs (Intel(R) Xeon(R) Platinum 8160 CPU @ 2.1 GHz) and 1488 GiB RAM was assigned to this machine. For ^177^Lu, the highest computing time and the highest RAM usage were observed for the 3D cell arrangement, cell geometry 4, membrane-bound internalization scenario and 5000 source points per cell, since the number of geometry components to be built and the number of source points were the largest in this case. A single simulation required 34 h for initialization, 111 h to 139 h for execution and 46 GiB RAM (measured for history 1). For ^225^Ac, the simulation with the highest computational cost (3D, geometry 1, membrane-bound, 10 source points per cell) required 1 h for initialization, 35 h to 85 h execution time depending on the number of alpha particles that hit the target (nucleus of the central cell) and 13 GiB RAM (measured for history 1). Simulations with a lower computational cost were run on two identical virtual machines with 12 vCPUs (Intel(R) Xeon(R) Silver 4210R CPU @ 2.4 GHz) and 78 GiB RAM each.

**Uncertainty of RBE**

The uncertainty of RBE was calculated based on the propagation of uncertainty:

$$\frac{\partial{RBE}_{{}^{225}{Ac}}}{\partial b_{{}^{225}{Ac}}}(D_{{}^{177}{Lu}})= \frac{{RBE}_{{}^{225}{Ac}}}{b_{{}^{225}{Ac}}}$$

$$\frac{\partial{RBE}_{{}^{225}{Ac}}}{\partial b_{{}^{177}{Lu}}}\left( D_{{}^{177}{Lu}} \right)=- \frac{{{RBE}_{{}^{225}{Ac}}}^{2}}{b_{{}^{225}{Ac}}}$$

$$\frac{\partial{RBE}_{{}^{225}{Ac}}}{\partial a_{{}^{177}{Lu}}}\left( D_{{}^{177}{Lu}} \right)=- \frac{D_{{}^{177}{Lu}} {{RBE}_{{}^{225}{Ac}}}^{2}}{b_{{}^{225}{Ac}}}$$

$$u\left( {RBE}_{{}^{225}{Ac}}\left( D_{{}^{177}{Lu}} \right) \right)= \sqrt{\left[ \frac{\partial{RBE}_{{}^{225}{Ac}}}{\partial b_{{}^{225}{Ac}}}\left( D_{{}^{177}{Lu}} \right)u(b_{{}^{225}{Ac}}) \right]^{2}+ \left[ \frac{\partial{RBE}_{{}^{225}{Ac}}}{\partial b_{{}^{177}{Lu}}}\left( D_{{}^{177}{Lu}} \right) u(b_{{}^{177}{Lu}}) \right]^{2}+ \left[ \frac{\partial{RBE}_{{}^{225}{Ac}}}{\partial a_{{}^{177}{Lu}}}\left( D_{{}^{177}{Lu}} \right) u(a_{{}^{177}{Lu}}) \right]^{2}}$$

$$\frac{\partial{RBE}_{{}^{225}{Ac}}}{\partial b_{{}^{225}{Ac}}}(D_{{}^{225}{Ac}})= b_{{}^{177}{Lu}}^{-1}\left( 1+4 \frac{b_{{}^{225}{Ac}}}{b_{{}^{177}{Lu}}} \frac{a_{{}^{177}{Lu}}}{b_{{}^{177}{Lu}}} D_{{}^{225}{Ac}} \right)^{-\frac{1}{2}}$$

$$\frac{\partial{RBE}_{{}^{225}{Ac}}}{\partial b_{{}^{177}{Lu}}}\left( D_{{}^{225}{Ac}} \right)=-2b_{{}^{225}{Ac}} \frac{\left( b_{{}^{177}{Lu}}^{2}+4a_{{}^{177}{Lu}}b_{{}^{225}{Ac}}D_{{}^{225}{Ac}} \right)^{-\frac{1}{2}}+1}{\left[ \left( b_{{}^{177}{Lu}}^{2}+4a_{{}^{177}{Lu}}b_{{}^{225}{Ac}}D_{{}^{225}{Ac}} \right)^{\frac{1}{2}}+b_{{}^{177}{Lu}} \right]^{2}}$$

$$\frac{\partial{RBE}_{{}^{225}{Ac}}}{\partial a_{{}^{177}{Lu}}}\left( D_{{}^{225}{Ac}} \right)=-4b_{{}^{225}{Ac}}^{2}D_{{}^{225}{Ac}} \frac{\left( b_{{}^{177}{Lu}}^{2}+4a_{{}^{177}{Lu}}b_{{}^{225}{Ac}}D_{{}^{225}{Ac}} \right)^{-\frac{1}{2}}}{\left[ \left( b_{{}^{177}{Lu}}^{2}+4a_{{}^{177}{Lu}}b_{{}^{225}{Ac}}D_{{}^{225}{Ac}} \right)^{\frac{1}{2}}+b_{{}^{177}{Lu}} \right]^{2}}$$

$$u\left( {RBE}_{{}^{225}{Ac}}\left( D_{{}^{225}{Ac}} \right) \right)= \sqrt{\left[ \frac{\partial{RBE}_{{}^{225}{Ac}}}{\partial b_{{}^{225}{Ac}}}\left( D_{{}^{225}{Ac}} \right)u(b_{{}^{225}{Ac}}) \right]^{2}+ \left[ \frac{\partial{RBE}_{{}^{225}{Ac}}}{\partial b_{{}^{177}{Lu}}}\left( D_{{}^{225}{Ac}} \right) u(b_{{}^{177}{Lu}}) \right]^{2}+ \left[ \frac{\partial{RBE}_{{}^{225}{Ac}}}{\partial a_{{}^{177}{Lu}}}\left( D_{{}^{225}{Ac}} \right) u(a_{{}^{177}{Lu}}) \right]^{2}}$$

**RBE as a function of absorbed dose**

The following inequality (note $D_{{}^{225}{Ac}}= D_{{}^{177}{Lu}}=D$)

$$\frac{\frac{b_{{}^{225}{Ac}}}{b_{{}^{177}{Lu}}}}{\frac{a_{{}^{177}{Lu}}}{b_{{}^{177}{Lu}}}D+1}>\frac{2b_{{}^{225}{Ac}}}{\sqrt{b_{{}^{177}{Lu}}^{2}+ +4a_{{}^{177}{Lu}}b_{{}^{225}{Ac}}D}+ b_{{}^{177}{Lu}}}$$

Is valid for absorbed doses to the nucleus satisfying the inequality

$b_{{}^{225}{Ac}}D > a_{{}^{177}{Lu}} D^{2}+ b_{{}^{177}{Lu}}D \underset{\Leftrightarrow}{} N_{DSB, {}^{225}{Ac}}>$ $N_{DSB, {}^{177}{Lu}}$


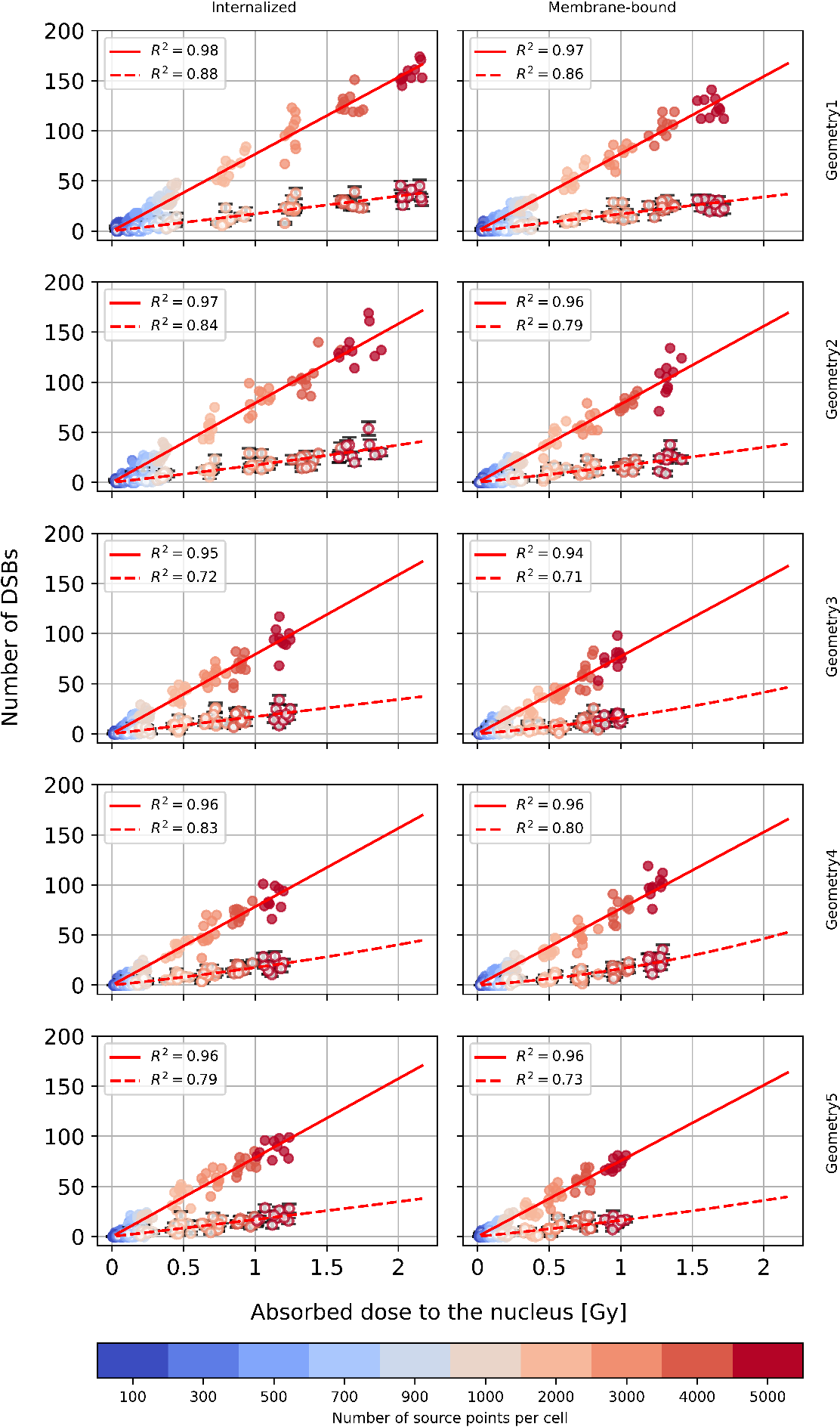


Figure 1: Simulated dose-effect curves for ^177^Lu, 2D cell arrangement sceanrio. Initial DSBs are shown as full circles, post-repair DSBs are depicted as circles with white background color. Error bars of number of DSBs after repair are shown in black.


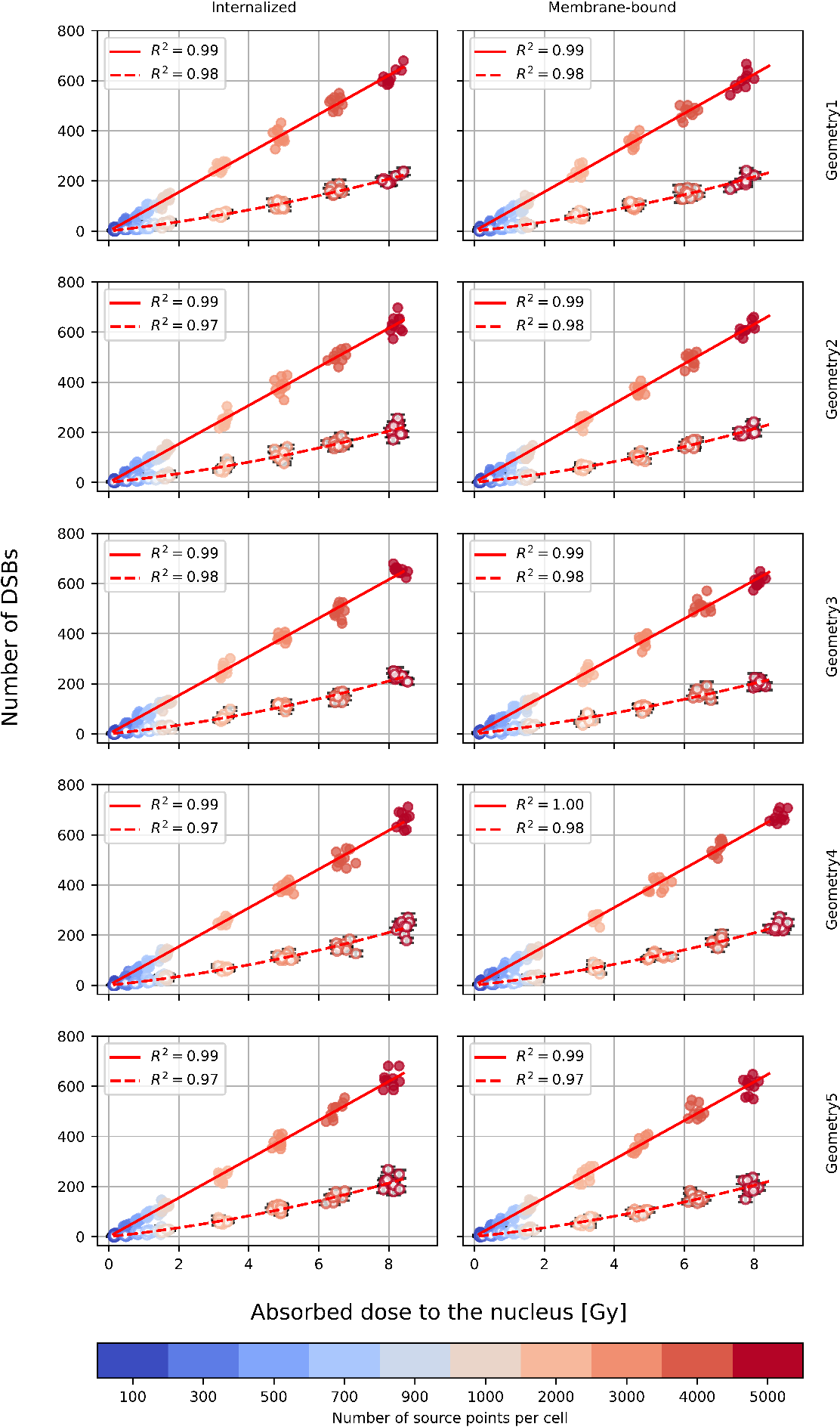


Figure 2: Simulated dose-effect curves for ^177^Lu, 3D cell arrangement sceanrio. Initial DSBs are shown as full circles, post-repair DSBs are depicted as circles with white background color. Error bars of number of DSBs after repair are shown in black.


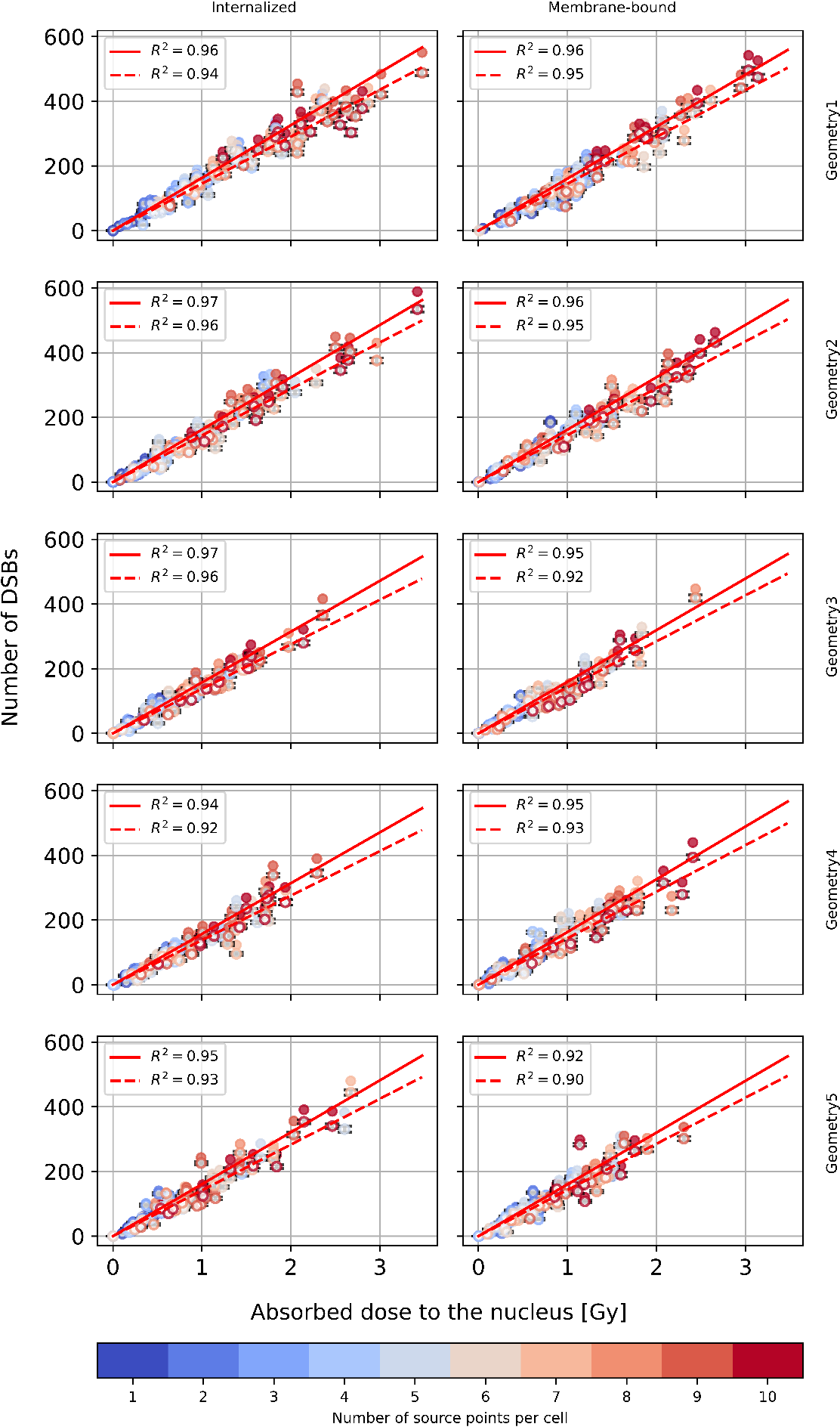


Figure 3: Simulated dose-effect curves for ^225^Ac, 2D cell arrangement sceanrio. Initial DSBs are shown as full circles, post-repair DSBs are depicted as circles with white background color. Error bars of number of DSBs after repair are shown in black.


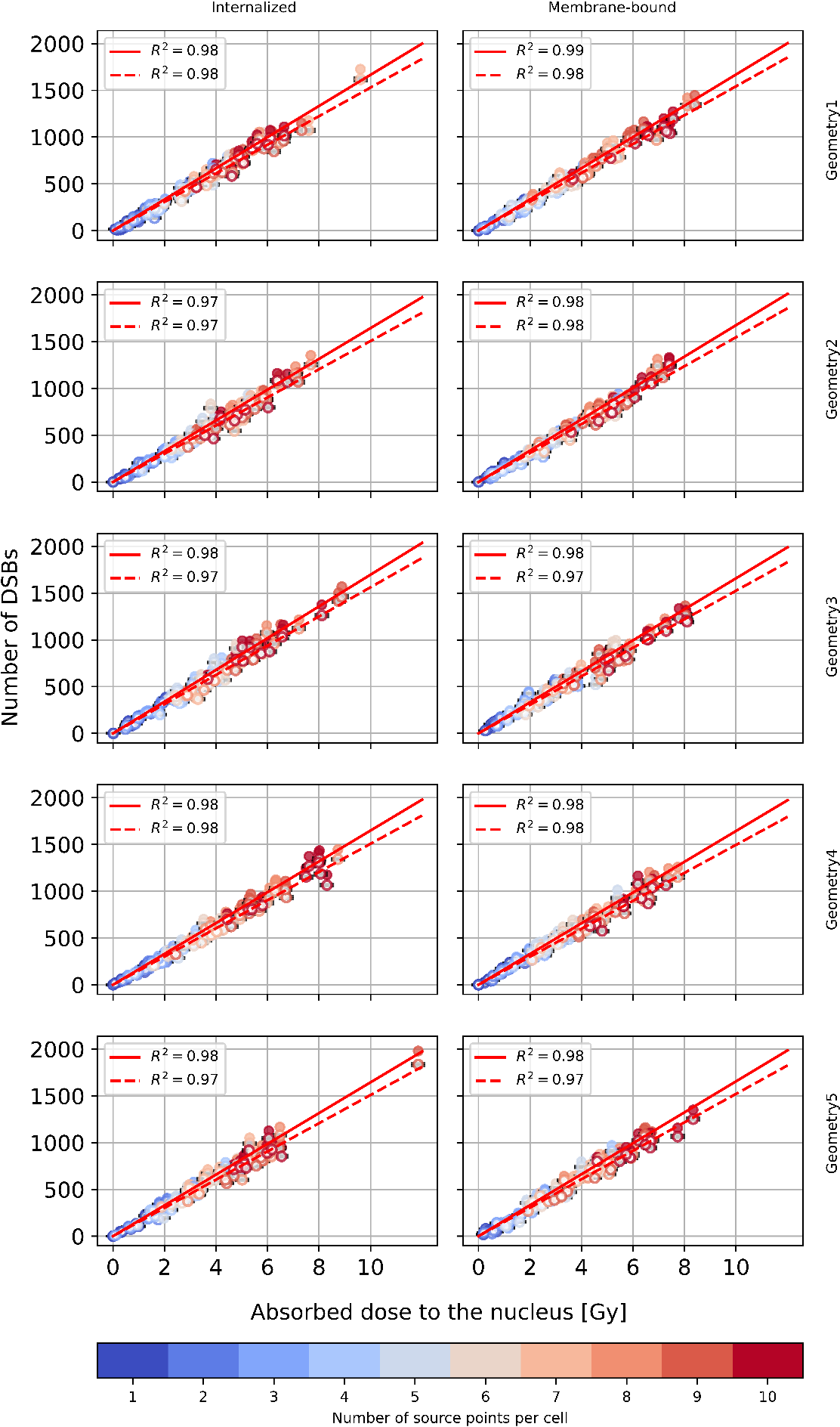


Figure 4: Simulated dose-effect curves for ^225^Ac, 3D cell arrangement sceanrio. Initial DSBs are shown as full circles, post-repair DSBs are depicted as circles with white background color. Error bars of number of DSBs after repair are shown in black.


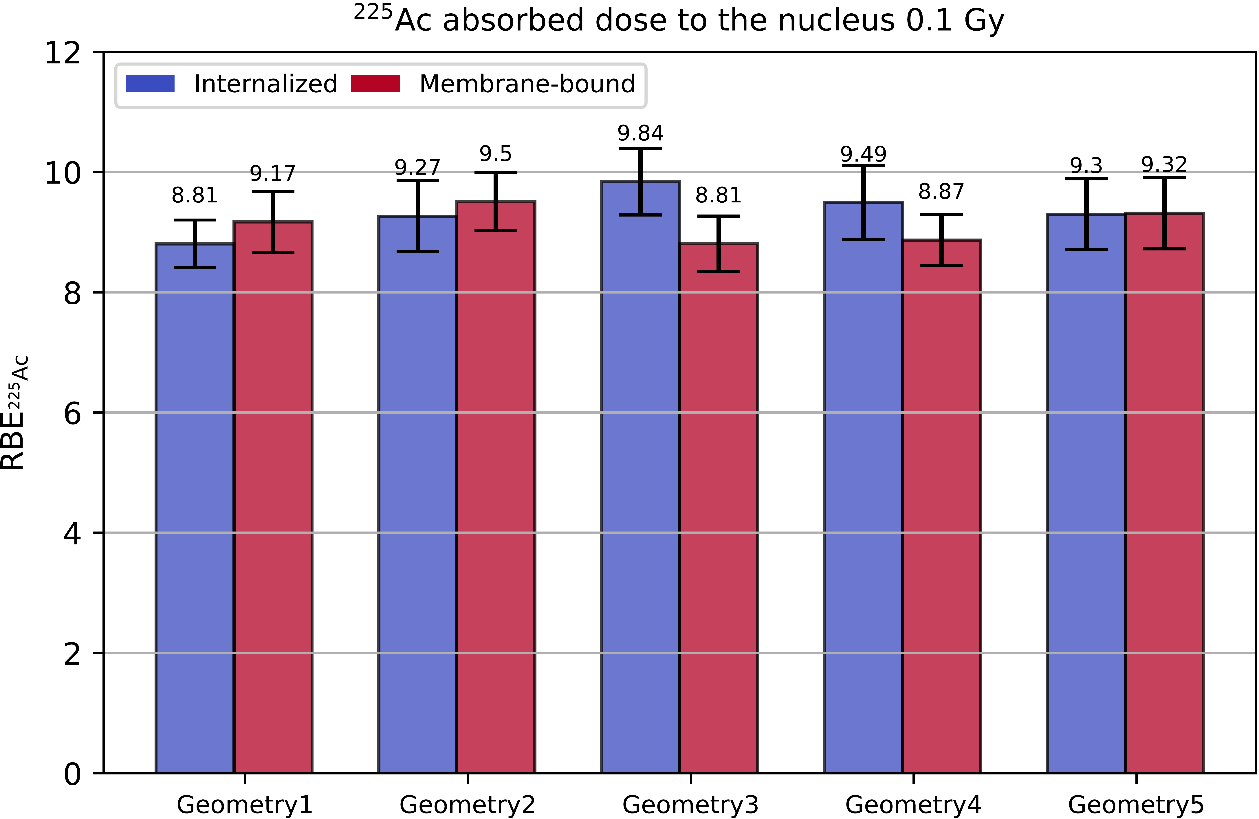


Figure 5: RBE of ^225^Ac at $D_{{}^{225}{Ac}} = 0.1 Gy$ based on the 3D data.


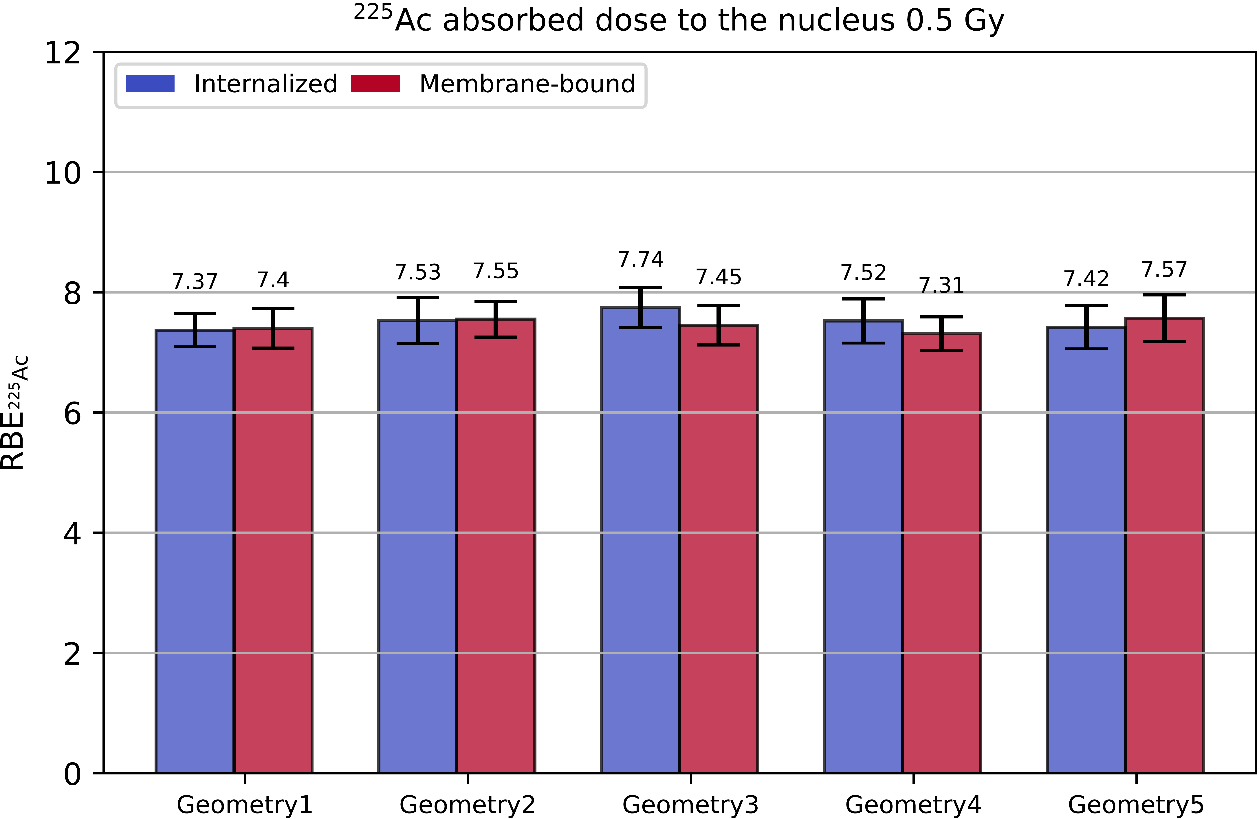


Figure 6: RBE of ^225^Ac at $D_{{}^{225}{Ac}} = 0.5 Gy$ based on the 3D data.


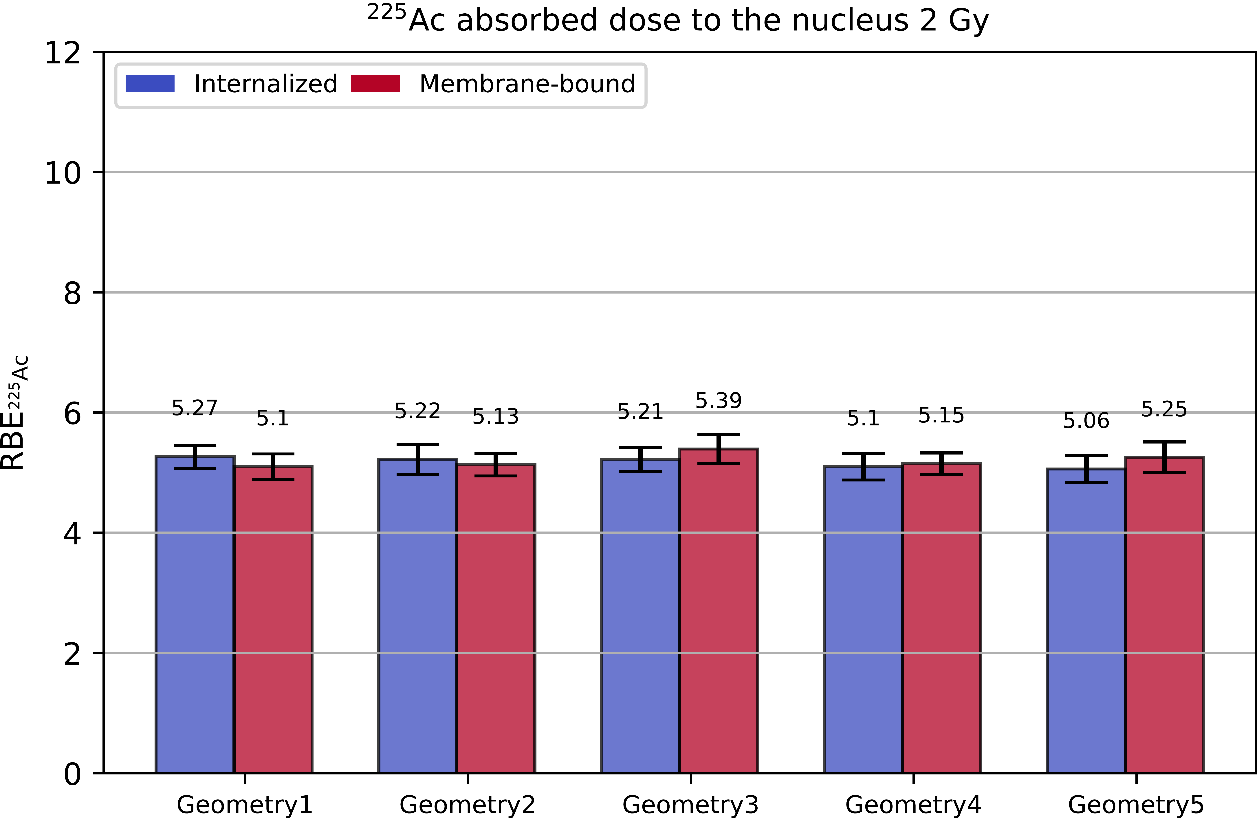


Figure 7: RBE of ^225^Ac at $D_{{}^{225}{Ac}} = 2 Gy$ based on the 3D data.


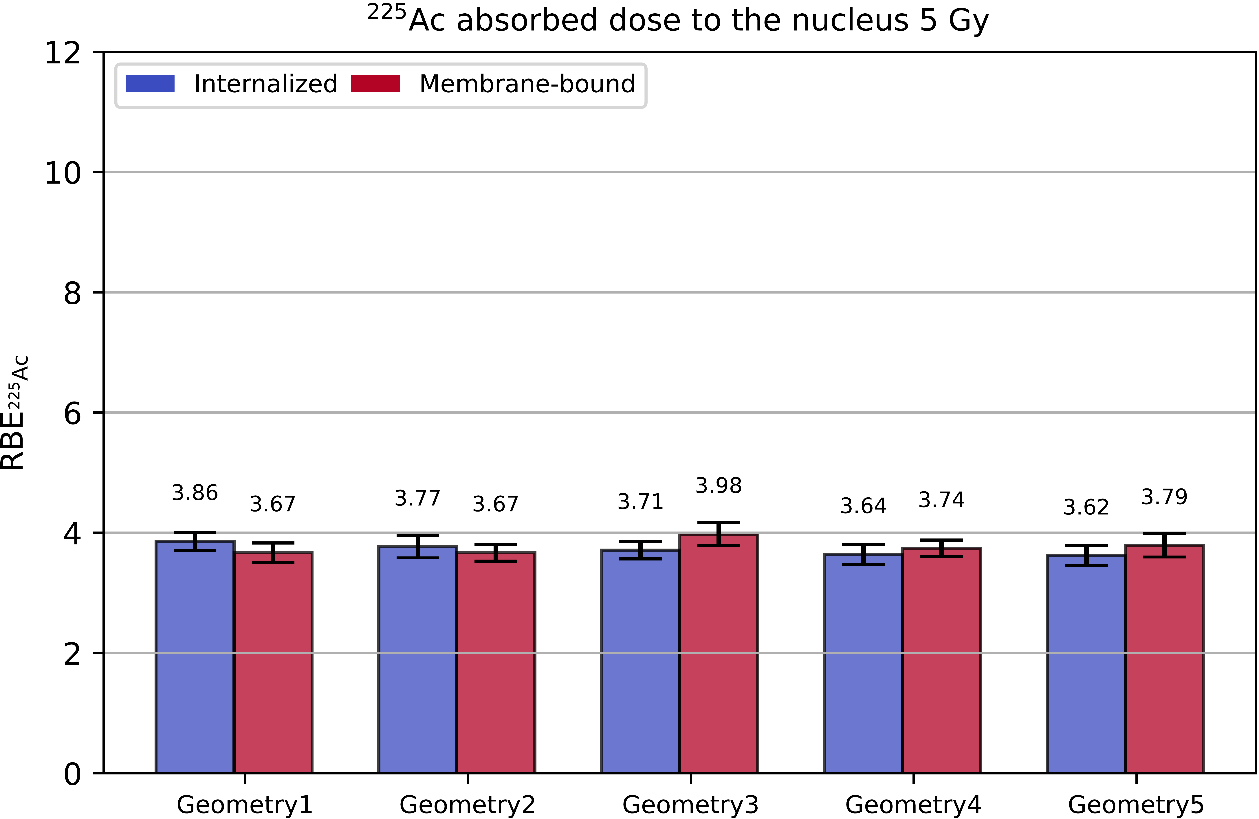


Figure 8: RBE of ^225^Ac at $D_{{}^{225}{Ac}} = 5 Gy$ based on the 3D data.


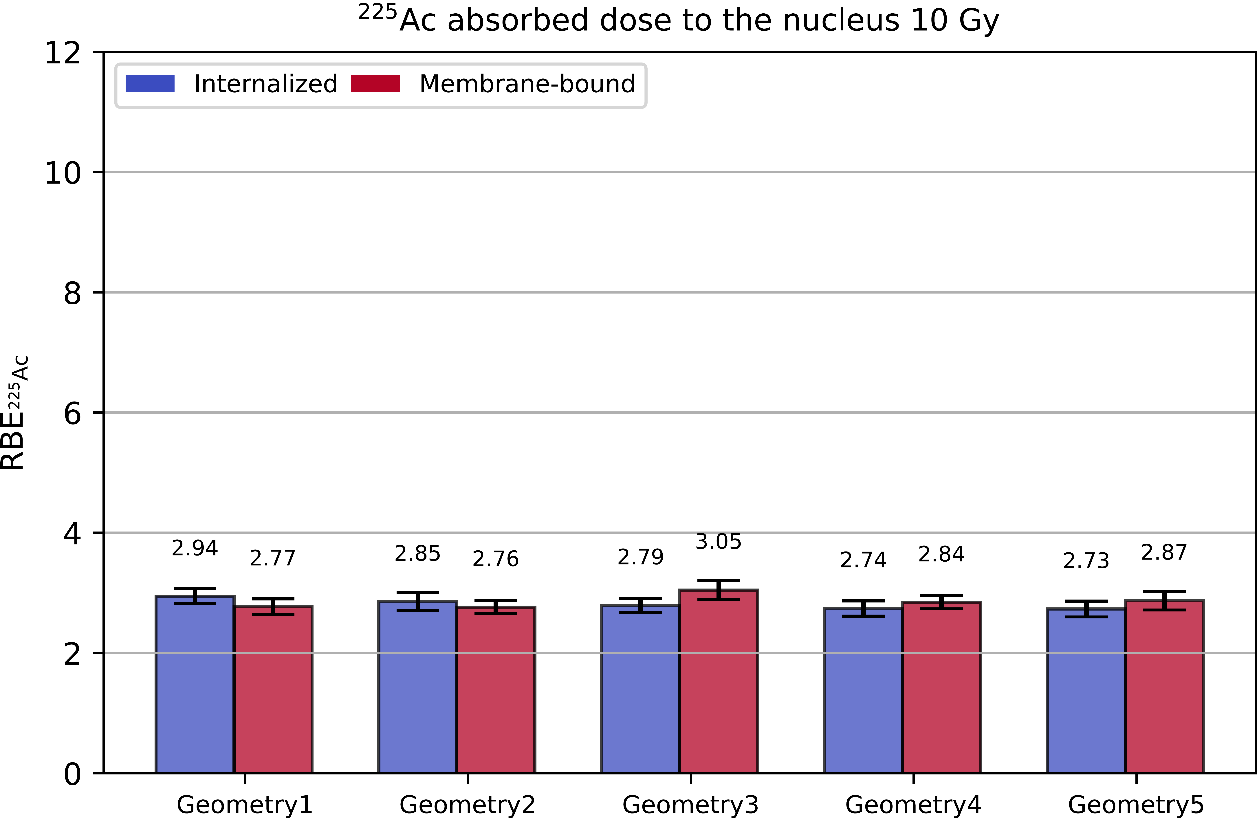


Figure 9: RBE of ^225^Ac at $D_{{}^{225}{Ac}} = 10 Gy$ based on the 3D data.


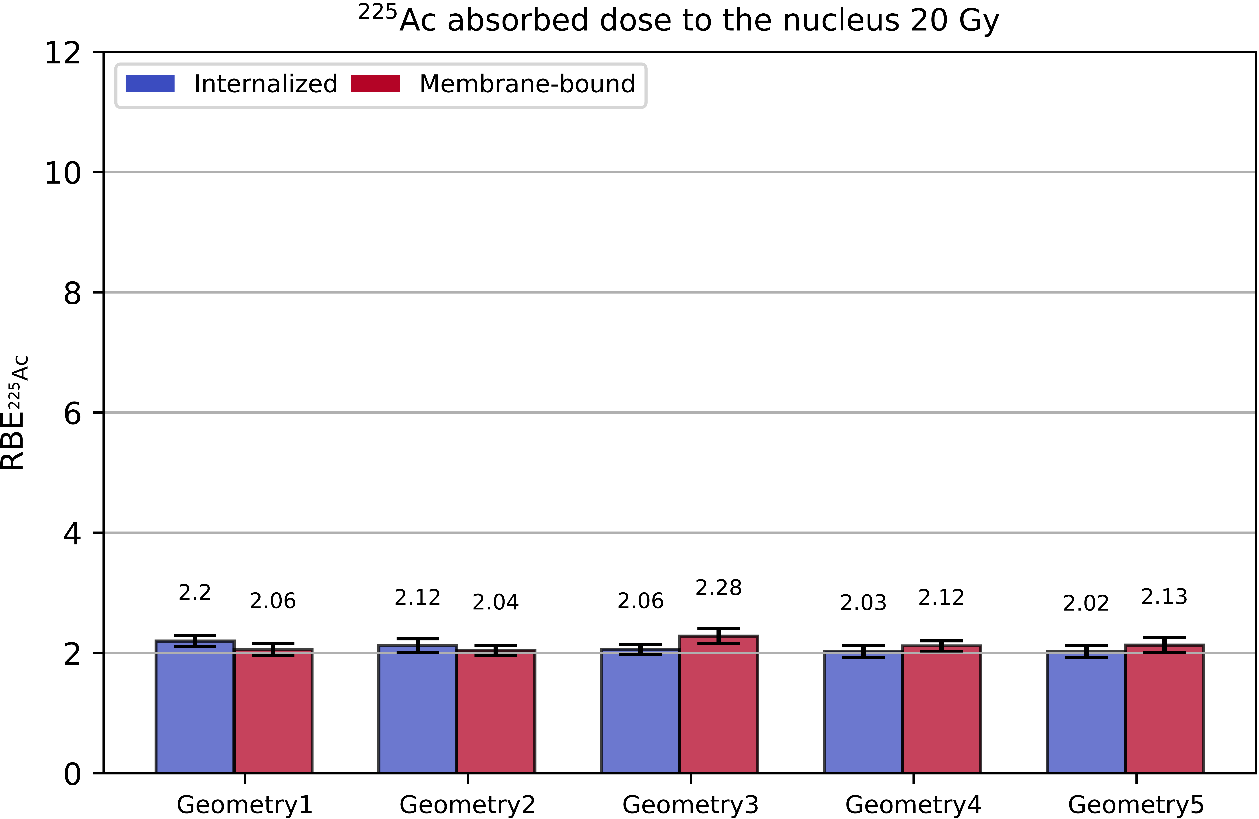


Figure 10: RBE of ^225^Ac at $D_{{}^{225}{Ac}} = 20 Gy$ based on the 3D data.


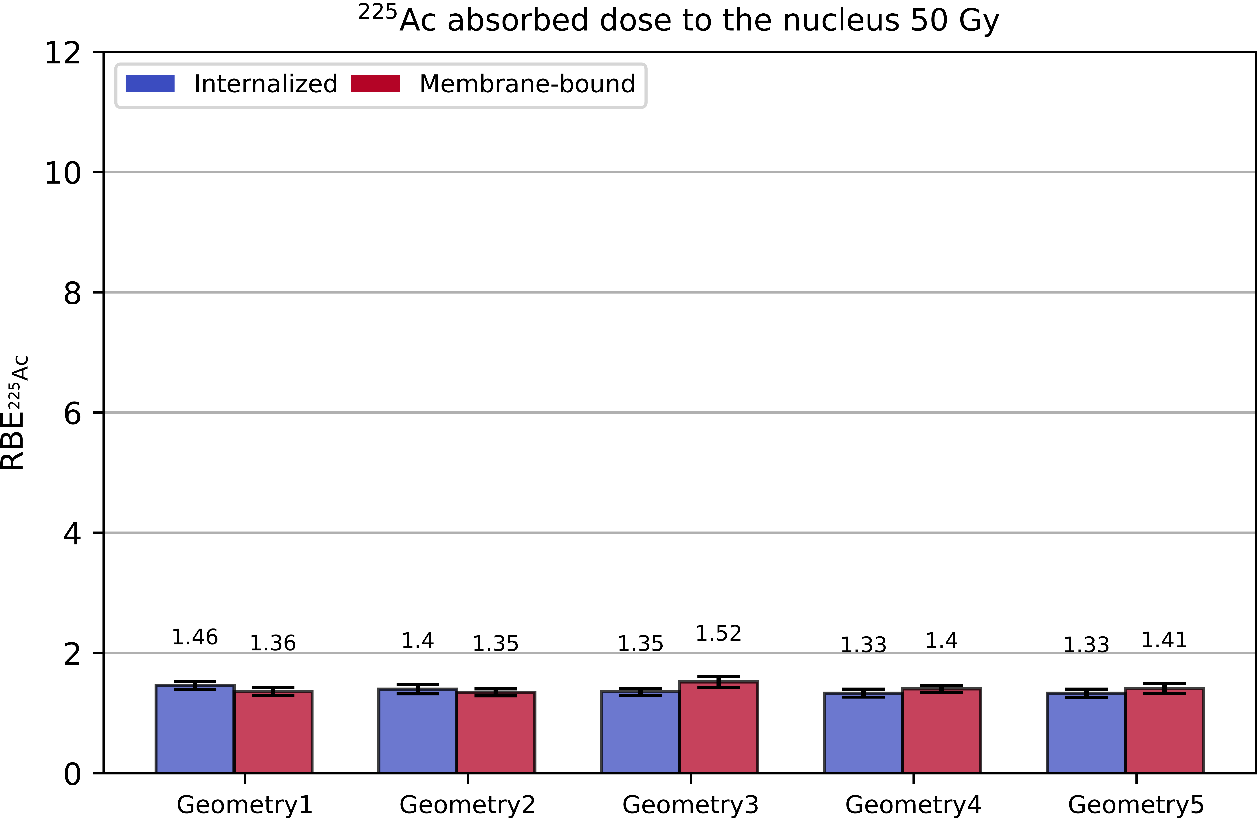


Figure 11: RBE of ^225^Ac at $D_{{}^{225}{Ac}} = 50 Gy$ based on the 3D data.


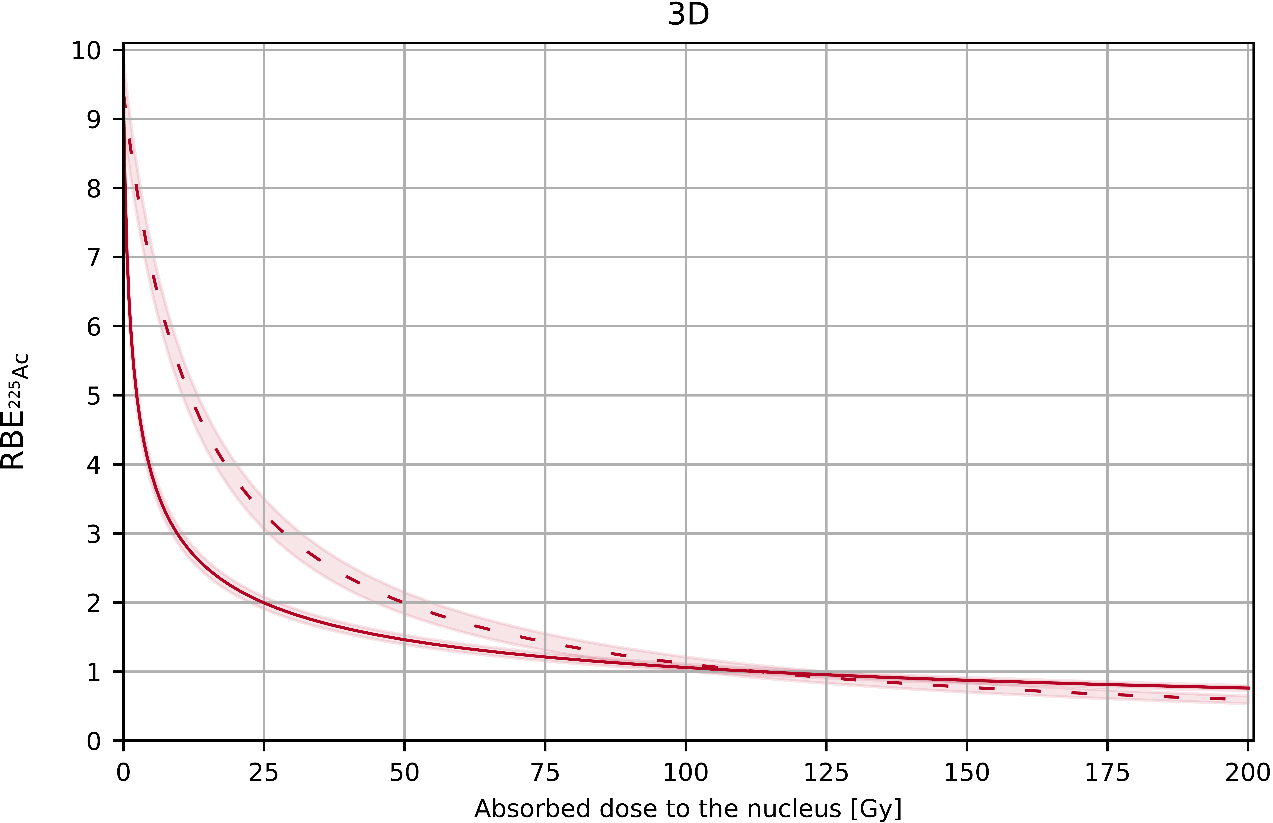


Figure 12: RBE of ^225^Ac as a function of the absorbed dose to the nucleus based on the post-repair damage for cell geometry 1 and full internalization. Dashed curve represents ${RBE}_{{}^{225}{Ac}}\left( D_{{}^{177}{Lu}} \right)$. ${RBE}_{{}^{225}{Ac}}\left( D_{{}^{225}{Ac}} \right)$ is shown with solid curve.

References:

1. Ramos-Méndez J, Schuemann J, Incerti S, Paganetti H, Schulte R, Faddegon B. Flagged uniform particle splitting for variance reduction in proton and carbon ion track-structure simulations. Physics in Medicine & Biology. 2017 Jul 6;62(15):5908.
2. Turner JE, Magee JL, Wright HA, Chatterjee A, Hamm RN, Ritchie RH. Physical and chemical development of electron tracks in liquid water. Radiation Research. 1983 Dec 1;96(3):437-49.
3. Bernal MA, Liendo JA. An investigation on the capabilities of the PENELOPE MC code in nanodosimetry. Medical physics. 2009 Feb;36(2):620-5.
4. Henthorn NT, Warmenhoven JW, Sotiropoulos M, Aitkenhead AH, Smith EA, Ingram SP, Kirkby NF, Chadwick AL, Burnet NG, Mackay RI, Kirkby KJ. Clinically relevant nanodosimetric simulation of DNA damage complexity from photons and protons. RSC advances. 2019;9(12):6845-58.
5. Schuemann J, McNamara AL, Warmenhoven JW, Henthorn NT, Kirkby KJ, Merchant MJ, Ingram S, Paganetti H, Held KD, Ramos-Mendez J, Faddegon B. A new standard DNA damage (SDD) data format. Radiation research. 2019 Jan;191(1):76-92.
6. Chang HH, Pannunzio NR, Adachi N, Lieber MR. Non-homologous DNA end joining and alternative pathways to double-strand break repair. Nature reviews Molecular cell biology. 2017 Aug;18(8):495-506.
